# Potent Cas9 Inhibition in Bacterial and Human Cells by AcrIIC4 and AcrIIC5 Anti-CRISPR Proteins

**DOI:** 10.1128/mBio.02321-18

**Published:** 2018-12-04

**Authors:** Jooyoung Lee, Aamir Mir, Alireza Edraki, Bianca Garcia, Nadia Amrani, Hannah E. Lou, Ildar Gainetdinov, April Pawluk, Raed Ibraheim, Xin D. Gao, Pengpeng Liu, Alan R. Davidson, Karen L. Maxwell, Erik J. Sontheimer

**Affiliations:** a RNA Therapeutics Institute, University of Massachusetts Medical School, Worcester, Massachusetts, USA; b Department of Biochemistry, University of Toronto, Toronto, Ontario, Canada; c Department of Molecular, Cell and Cancer Biology, University of Massachusetts Medical School, Worcester, Massachusetts, USA; d Department of Molecular Genetics, University of Toronto, Toronto, Ontario, Canada; e Program in Molecular Medicine, University of Massachusetts Medical School, Worcester, Massachusetts, USA; Max Planck Institute for Infection Biology; North Carolina State University; Université Laval

**Keywords:** CRISPR, Cas9, type II-C, anti-CRISPR, crRNA

## Abstract

In their natural settings, CRISPR-Cas systems play crucial roles in bacterial and archaeal adaptive immunity to protect against phages and other mobile genetic elements, and they are also widely used as genome engineering technologies. Previously we discovered bacteriophage-encoded Cas9-specific anti-CRISPR (Acr) proteins that serve as countermeasures against host bacterial immunity by inactivating their CRISPR-Cas systems (A. Pawluk, N. Amrani, Y. Zhang, B. Garcia, et al., Cell 167:1829–1838.e9, 2016, https://doi.org/10.1016/j.cell.2016.11.017). We hypothesized that the evolutionary advantages conferred by anti-CRISPRs would drive the widespread occurrence of these proteins in nature (K. L. Maxwell, Mol Cell 68:8–14, 2017, https://doi.org/10.1016/j.molcel.2017.09.002; A. Pawluk, A. R. Davidson, and K. L. Maxwell, Nat Rev Microbiol 16:12–17, 2018, https://doi.org/10.1038/nrmicro.2017.120; E. J. Sontheimer and A. R. Davidson, Curr Opin Microbiol 37:120–127, 2017, https://doi.org/10.1016/j.mib.2017.06.003). We have identified new anti-CRISPRs using the same bioinformatic approach that successfully identified previous Acr proteins (A. Pawluk, N. Amrani, Y. Zhang, B. Garcia, et al., Cell 167:1829–1838.e9, 2016, https://doi.org/10.1016/j.cell.2016.11.017) against Neisseria meningitidis Cas9 (NmeCas9). In this work, we report two novel anti-CRISPR families in strains of Haemophilus parainfluenzae and Simonsiella muelleri, both of which harbor type II-C CRISPR-Cas systems (A. Mir, A. Edraki, J. Lee, and E. J. Sontheimer, ACS Chem Biol 13:357–365, 2018, https://doi.org/10.1021/acschembio.7b00855). We characterize the type II-C Cas9 orthologs from H. parainfluenzae and S. muelleri, show that the newly identified Acrs are able to inhibit these systems, and define important features of their inhibitory mechanisms. The S. muelleri Acr is the most potent NmeCas9 inhibitor identified to date. Although inhibition of NmeCas9 by anti-CRISPRs from H. parainfluenzae and S. muelleri reveals cross-species inhibitory activity, more distantly related type II-C Cas9s are not inhibited by these proteins. The specificities of anti-CRISPRs and divergent Cas9s appear to reflect coevolution of their strategies to combat or evade each other. Finally, we validate these new anti-CRISPR proteins as potent off-switches for Cas9 genome engineering applications.

## Abstract (precis)

As one of their countermeasures against CRISPR-Cas immunity, bacteriophages have evolved natural inhibitors known as anti-CRISPR (Acr) proteins. Despite the existence of such examples for type II CRISPR-Cas systems, we currently know relatively little about the breadth of Cas9 inhibitors, and most of their direct Cas9 targets are uncharacterized. In this work we identify two new type II-C anti-CRISPRs and their cognate Cas9 orthologs, validate their functionality *in vitro* and in bacteria, define their inhibitory spectrum against a panel of Cas9 orthologs, demonstrate that they act before Cas9 DNA binding, and document their utility as off-switches for Cas9-based tools in mammalian applications. The discovery of diverse anti-CRISPRs, the mechanistic analysis of their cognate Cas9s, and the definition of Acr inhibitory mechanisms afford deeper insight into the interplay between Cas9 orthologs and their inhibitors and provide greater scope for exploiting Acrs for CRISPR-based genome engineering.

## INTRODUCTION

Clustered, regularly interspaced, short, palindromic repeats (CRISPRs) and their CRISPR-associated (*cas*) genes constitute a prokaryotic adaptive immune defense system against foreign genetic elements such as phages and plasmids (1–3). The components of CRISPR-Cas systems that allow recognition and destruction of invading genetic elements are extremely diverse and form the basis for the current CRISPR-Cas classification framework (4), which includes two broad classes, six major types, and many subtypes. In class 1 CRISPR-Cas systems, effector modules form a multiprotein complex, whereas class 2 systems use a single effector protein to target foreign nucleic acids. Cas9 is an effector protein in the best-characterized class 2 system, type II, which is further divided into three subtypes (II-A, -B, and -C) based on Cas9 phylogeny and the presence or absence of additional adaptation-related Cas proteins (4). Cas9 is a single-component, RNA-guided endonuclease that employs the CRISPR RNA (crRNA) as a sequence-specific guide to target foreign DNA (5), with the help of a *trans*-activating RNA (tracrRNA) (6), which can be fused to the crRNA to form a single guide RNA (sgRNA) (7). The robustness and ease of Cas9 programmability have greatly facilitated its rapid adoption in genome editing and modulation (8).

Although Cas9s have attracted unprecedented attention for genome engineering applications, their natural function in bacterial defense plays a crucial role in the ongoing battle against phages and other invading mobile genetic elements (MGEs). As countermeasures against such a powerful barrier, phages and MGEs have evolved numerous, distinct strategies to overcome bacterial defenses (9). Anti-CRISPR (Acr) proteins provide one way to directly disarm CRISPR-Cas systems. The existence of Acrs was first shown in phages that successfully infect Pseudomonas aeruginosa strains despite the presence of active type I CRISPR-Cas systems and matching CRISPR spacers (10). The sixteen reported type I Acr families (11–13) do not share common structural similarities or sequences but are frequently encoded adjacent to putative transcriptional regulator genes known as anti-CRISPR-associated (*aca*) genes (14). The first type II-specific *acr* genes were identified as previously uncharacterized open reading frames (ORFs) adjacent to predicted *aca* genes in MGEs of bacteria harboring type II CRISPR-Cas systems (15). Additional Acrs have been found by identifying candidate *acr* genes in lysogens embedded within genomes harboring potentially self-targeting type II CRISPR-Cas systems (16), or by screening lytic phages for the ability to resist type II CRISPR defenses (17, 18). Type V anti-CRISPRs have also been discovered recently (13, 19). Type II and type V Acrs are of particular interest because they can potentially provide temporal, spatial, or conditional control over Cas9- and Cas12a-based applications.

Thus far, three families of type II-C Acrs (15) and six families of type II-A Acrs (16–18) have been reported, and inhibitory mechanisms are known in a few cases (15, 16, 20). For instance, AcrIIA4*_Lmo_*, a type II-A Acr that can inhibit the most widely used Cas9 ortholog from Streptococcus pyogenes (SpyCas9), prevents Cas9 DNA binding (16) by occupying the protospacer adjacent motif (PAM)-interacting domain (PID) and masking the RuvC nuclease domain, in part via DNA mimicry (21–23). Conversely, a type II-C Acr, AcrIIC1*_Nme_*, does not prevent target DNA binding by Neisseria meningitidis Cas9 (NmeCas9, from strain 8013), but rather binds and inhibits the enzyme’s HNH nuclease domain (20). Yet another type II-C Cas9 inhibitor, AcrIIC3*_Nme_*, prevents target DNA binding (15) in a manner that is accompanied by NmeCas9 dimerization (20).

Because Acrs provide obvious fitness advantages (24) to phages and MGEs that must counteract a diversity of CRISPR-Cas systems, we hypothesized that many more type II Acrs likely remain to be discovered. Here, we identify two new type II-C Cas9 inhibitors from strains of H. parainfluenzae (AcrIIC4*_Hpa_*) and S. muelleri (AcrIIC5*_Smu_*). We characterize their cognate Cas9 proteins from H. parainfluenzae and S. muelleri and show that these proteins are functional *in vivo* and *in vitro*. Further, we show that AcrIIC5*_Smu_* is the most potent NmeCas9 inhibitor reported to date. While both of these Acrs inhibit DNA binding by Cas9, including during mammalian genome editing applications, they differ in their phylogenetic ranges of Cas9 inhibition.

## RESULTS

### Identification of novel type II-C anti-CRISPR proteins.

**FIG 1:**
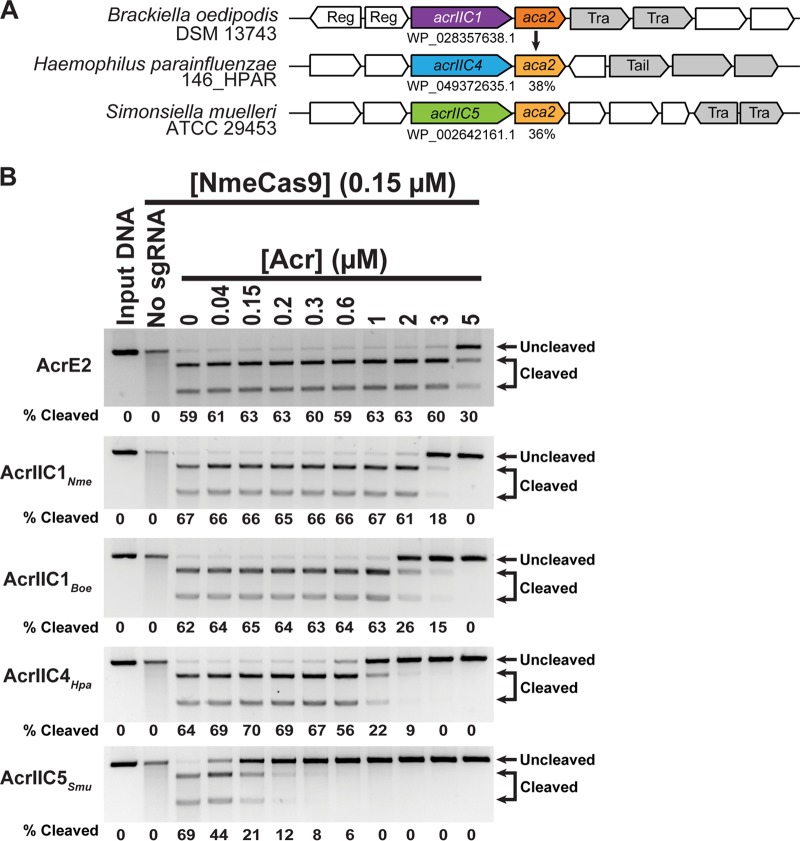
Identification and *in vitro* validation of two anti-CRISPR protein families. (A) Schematic of candidate anti-CRISPR proteins and *aca2* genes in the genomic context of H. parainfluenzae (AcrIIC4*_Hpa_*) and S. muelleri (AcrIIC5*_Smu_*). Gray genes are associated with mobile DNA, and known gene functions are annotated as follows: “Reg” is a transcriptional regulator, “Tail” is involved in phage tail morphogenesis, and “Tra” is a transposase. The B. oedipodis *aca2* gene is used as a query for pBLAST searches, and percent identities of *aca2* orthologs are denoted. Arrows are not drawn to scale. (B) *In vitro* cleavage of target DNA by the NmeCas9-sgRNA complex in the presence of anti-CRISPR protein. Preformed NmeCas9-sgRNA RNP complex was incubated with purified anti-CRISPR proteins as indicated with AcrE2 as a negative control, AcrIIC1 as a positive control, and candidate Acrs. Then, a linearized plasmid with a protospacer and PAM sequence was added to the reaction mixture. Molarities of anti-CRISPR protein (relative to constant Cas9 molarity) are shown at the top of each lane, mobilities of input and cleaved DNAs are denoted on the right, and cleavage efficiencies (“% cleaved”) are given at the bottom of each lane.

We previously developed a “guilt-by-association” bioinformatics approach that allowed the identification of novel families of anti-CRISPR proteins encoded in phages and MGEs of diverse bacterial species (14, 15, 25). In this pipeline, new *acr* gene candidates are identified by their proximity to predicted helix-turn-helix (HTH) transcriptional regulator genes known as *aca* genes. We began BLASTp searches with the *aca2* gene (WP_028357637.1) from Brackiella oedipodis DSM 13743 (NZ_KK211205.1) immediately downstream of AcrIIC1*_Boe_* (WP_028357638.1) (15). We focused specifically on hits in genomes belonging to species in which type II-C CRISPR-Cas systems are encoded, reasoning that mobile genetic elements in those genomes would be most likely to encode anti-CRISPR activity against the type II-C Cas9 of their host. We identified ORFs encoding uncharacterized small (∼50- to 150-amino-acid [aa]) proteins immediately upstream of *aca2* orthologs, focusing on genomic regions near putative phage- or MGE-associated sequences (11, 26, 27). These criteria led us to focus on two putative Acr candidates: an 88-aa hypothetical protein in the genome of H. parainfluenzae strain 146_HPAR (WP_049372635.1) and a 130-aa hypothetical protein in the genome of S. muelleri strain ATCC 29453 (WP_002642161.1; see Table S1 in the supplemental material). Both are located upstream of apparent *aca2* orthologs of H. parainfluenzae and S. muelleri, and these orthologs share 38% (WP_049372634.1) and 36% (WP_002642160.1) identity to B. oedipodis *aca2*, respectively, and 51% identity to each other (Fig. 1A). AcrIIC4 has only one detectable homolog of 97% identity in a different strain of H. parainfluenzae, and AcrIIC5 has distant orthologs in *Neisseria* species with ∼30% identity (Table S2). Both strains encode predicted type II-C CRISPR-Cas machineries with Cas9 orthologs that exhibit 59% and 62% identity with NmeCas9, respectively (Table S3). Based on these similarities, the previously established abilities of some type II anti-CRISPRs to inhibit Cas9 orthologs outside their host species (15–18, 20), and the existence of apparent orthologs of the S. muelleri candidate Acr in multiple examples from *Neisseria* (Table S2), we first tested for anti-CRISPR activity against the well-characterized NmeCas9. We cloned each candidate Acr sequence into a bacterial expression vector, purified recombinant proteins from Escherichia coli, and tested their abilities to prevent DNA cleavage by NmeCas9 *in vitro* (Fig. 1B). When each of the purified candidate Acrs was added to parallel reaction mixtures, cleavage was inhibited in a concentration-dependent manner, with complete inhibition being reached at ∼20-fold (H. parainfluenzae candidate) and ∼7-fold (S. muelleri candidate) molar excess of Acr (Fig. 1B). Incubation with AcrE2, an 84-aa type I-E anti-CRISPR (14, 25) included as a negative control, did not affect target DNA cleavage by NmeCas9. When we compared the ability of Acrs to inhibit DNA cleavage when first added to the apo or sgRNA-loaded forms of NmeCas9, both candidate Acrs inhibited the two forms of NmeCas9 to a comparable extent (Fig. S1). This observation is in contrast to previously described orthologous anti-CRISPRs AcrIIC1*_Boe_* and AcrIIC1*_Nme_*, which were less potent when added to the NmeCas9:sgRNA complex (Fig. S1). Because these initial tests confirmed the anti-CRISPR activities of the two candidates from H. parainfluenzae and S. muelleri, we named them AcrIIC4*_Hpa_* and AcrIIC5*_Smu_*, respectively, to conform with established Acr nomenclature (11, 25).

10.1128/mBio.02321-18.1 FIG S1 Inhibition of apoNmeCas9 cleavage *in vitro* of target DNA. The experiment was as in Fig. 1B, but with Acrs incubated with apoCas9 before adding sgRNA and target DNA. Stoichiometries of anti-CRISPR proteins (relative to constant Cas9 molarity) are shown at the top of each lane, mobilities of input and cleaved DNAs are denoted on the right, and cleavage efficiencies (“% cleaved”) are given at the bottom of each lane. Download FIG S1, PDF file, 12.5 MB. Copyright © 2018 Lee et al. 2018 Lee et al. This content is distributed under the terms of the Creative Commons Attribution 4.0 International license.

10.1128/mBio.02321-18.7 TABLE S1 DNA and amino acid sequences of newly identified II-C anti-CRISPRs and Cas9 orthologs. Download Table S1, PDF file, 0.05 MB. Copyright © 2018 Lee et al. 2018 Lee et al. This content is distributed under the terms of the Creative Commons Attribution 4.0 International license.

10.1128/mBio.02321-18.8 TABLE S2 AcrIIC5_*Smu*_ homolog % identities. Download Table S2, PDF file, 0.02 MB. Copyright © 2018 Lee et al. 2018 Lee et al. This content is distributed under the terms of the Creative Commons Attribution 4.0 International license.

10.1128/mBio.02321-18.9 TABLE S3 Pairwise percent protein identities between type II-C Cas9 orthologs. Download Table S3, PDF file, 0.02 MB. Copyright © 2018 Lee et al. 2018 Lee et al. This content is distributed under the terms of the Creative Commons Attribution 4.0 International license.

### H. parainfluenzae and S. muelleri encode type II-C CRISPR-Cas systems that function *in vitro*.

**FIG 2:**
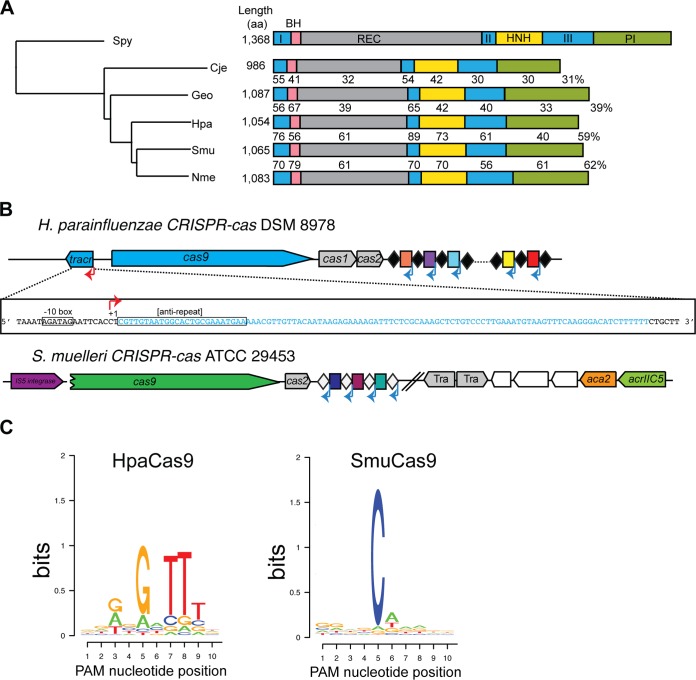
Characterization of new type II-C Cas9 orthologs. (A) A phylogenetic tree of type II Cas9 orthologs from S. pyogenes, N. meningitidis, C. jejuni, G. stearothermophilus, H. parainfluenzae, and S. muelleri. Domains are drawn to scale and colored as follows: blue, RuvC-I, -II, and -III nuclease domain; pink, bridge-helix (BH); gray, recognition lobe (REC); yellow, HNH nuclease domain; green, PAM-interacting domain (PI). Percent identities of type II-C orthologs to NmeCas9 are indicated. (B) Genomic architectures of CRISPR-*cas* loci of H. parainfluenzae DSM 8978 and S. muelleri ATCC 29453. The sequence of the HpaCas9 tracrRNA is shown in the inset. Individual genomic elements are not drawn to scale. (C) PAM preferences for H. parainfluenzae (left) and S. muelleri (right) Cas9 proteins. Frequencies of nucleotides at each PAM position were calculated and plotted as a WebLogo.

Anti-CRISPRs are most likely to inhibit the Cas9 ortholog expressed by the same species, but to our knowledge, little was known about the Cas9 orthologs from H. parainfluenzae and S. muelleri. To address this, we characterized these type II-C CRISPR-Cas systems (Fig. 2A). First, we identified a 1,054-aa *cas9* ORF in H. parainfluenzae DSM 8978, a strain closely related to H. parainfluenzae 146_HPAR for which genomic DNA sequence was available. We identified a predicted tracrRNA adjacent to *cas9* and noted that the CRISPR repeat sequence included a likely minimal promoter that initiates transcription in the flanking spacer, as found previously with other type II-C systems (28) (Fig. 2B). The predicted transcriptional start site would yield a crRNA with a 24-nt spacer, similar to N. meningitidis strain 8013 (29). We then used tracrRNA:crRNA complementarity to predict an sgRNA scaffold (Fig. S2A). These components were then used to generate expression constructs for recombinant HpaCas9 in E. coli, and for its sgRNA via *in vitro* transcription, for biochemical analyses (see below).

10.1128/mBio.02321-18.2 FIG S2 Characterization of new type II-C Cas9 orthologs. (A) Predicted crRNA:tracrRNA structures for NmeCas9 and HpaCas9. Nucleotides that are different between the two orthologs are underlined. (B) Phage and plasmid targets matching H. parainfluenzae spacer sequences. The PAM region is highlighted in yellow. (C) Breadth of inhibition of NmeCas9, GeoStCas9, GeoL300Cas9, and CjeCas9. The double asterisk denotes sgRNA. Download FIG S2, PDF file, 21.1 MB. Copyright © 2018 Lee et al. 2018 Lee et al. This content is distributed under the terms of the Creative Commons Attribution 4.0 International license.

Unlike H. parainfluenzae DSM 8978, the CRISPR-*cas* locus of S. muelleri ATCC 29453 appeared to be degenerate (Fig. 2B). There is no apparent *cas1*, and the *cas2* lacks a canonical ATG start codon. However, the *cas9* ORF (1,065 aa) is intact and has all the predicted functional domains found in other Cas9 orthologs, which suggested that SmuCas9 itself might be active. When we attempted to define an appropriate guide RNA scaffold for SmuCas9, we could not predict its tracrRNA (based in part on crRNA complementarity) from nearby genomic sequences. Instead, we found an IS*5* integrase upstream of *cas9*, where a tracrRNA locus is often observed (Fig. 2B). Although we sequenced ∼2 kb flanking the CRISPR locus to fill gaps in the genome assembly, we could not detect a tracrRNA sequence. As an alternative, we took advantage of the nonorthogonality of sgRNAs to closely related Cas9 orthologs (30, 31) and used the NmeCas9 sgRNA to test the cleavage activity of SmuCas9.

**FIG 3:**
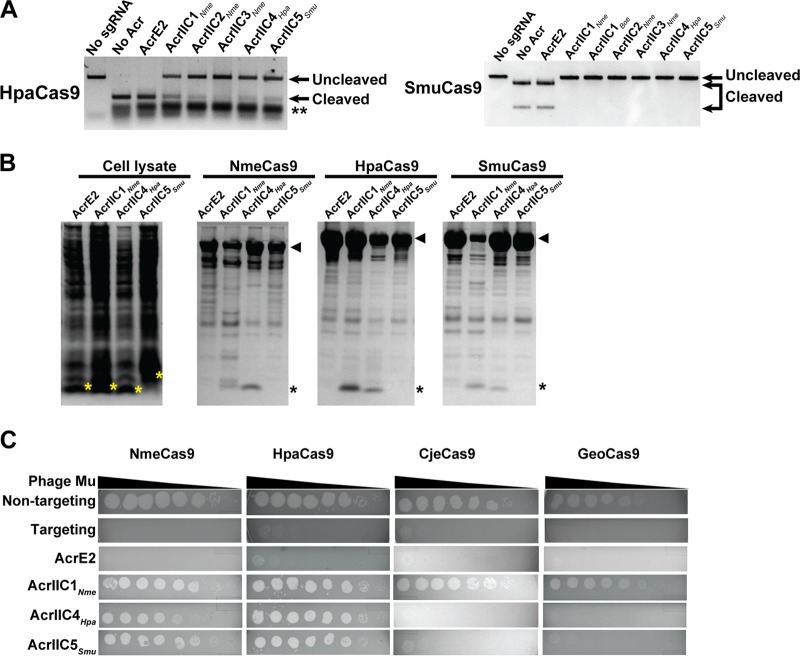
Validation of Cas9 and anti-CRISPR proteins from H. parainfluenzae and S. muelleri. (A) Validation of HpaCas9 and SmuCas9 cleavage activity and inhibition by anti-CRISPR proteins *in vitro*. The double asterisk denotes sgRNA. (B) Interaction between Acrs and NmeCas9, HpaCas9, and SmuCas9 is visualized by Coomassie blue staining after copurification of each 6×His-tagged Cas9 and untagged Acrs from E. coli. Each Cas9 ortholog and anti-CRISPRs are indicated as arrowheads and asterisks, respectively. (C) Plaquing of E. coli phage Mu targeted by the Nme, Hpa, Cje, or Geo Cas9 in the presence of the anti-CRISPR proteins. Tenfold serial dilutions of phage Mu lysate were spotted on lawns of bacteria expressing the indicated Acr proteins. Data are from one plate representative of ≥3 replicates.

To define the PAM requirements for HpaCas9 and SmuCas9, a library of short DNA fragments containing a unique protospacer flanked by 10-nt randomized PAM sequences was subjected to *in vitro* digestion using purified, recombinant Cas9 proteins and T7-transcribed sgRNAs. Next, digested products were gel purified and deep sequenced. PAM sequences were identified from the resulting sequencing data based on the frequency of nucleotides at each position of the digested products. HpaCas9 had strong preference for 5′-N_4_GNTT-3′ PAM sequence (Fig. 2C). Notably, this PAM is similar to the consensus PAM sequence of NmeCas9 (29, 31–33, 47). We extracted spacer sequences from the H. parainfluenzae 146_HPAR CRISPR locus and identified two candidate protospacers (Fig. S2B). When we aligned the nucleotide sequences adjacent to the protospacers, we noted that both contained a 5′-N_4_GATT-3′, which is consistent with the PAM discovered *in vitro* (Fig. 2C and Fig. S2B). We found that SmuCas9 had strong preference for the 5′-N_4_C-3′ PAM sequence (Fig. 2C). This single cytosine at the 5th position from the protospacer appears to be the most critical PAM nucleotide by far, although moderate preferences for other nucleotides at other positions cannot be excluded from this analysis. We validated these putative PAMs by performing *in vitro* cleavage of a nondegenerate substrate and confirmed efficient cleavage of a DNA target bearing a 5′-N_4_GNTT-3′ PAM for HpaCas9 and a 5′-N_4_C-3′ PAM for SmuCas9 (Fig. 3A).

### AcrIIC4*_Hpa_* and AcrIIC5*_Smu_* inhibit their native, cognate Cas9 proteins and close orthologs *in vitro* and in bacteria.

We next examined the ability of AcrIIC4*_Hpa_* and AcrIIC5*_Smu_* to inhibit HpaCas9 and SmuCas9, which share 59% and 62% sequence identity with NmeCas9, respectively (Fig. 2A and Fig. S3). Our *in vitro* DNA cleavage analyses show that these Acrs can inactivate their cognate Cas9 proteins (Fig. 3A). Given that some type II Acrs can inhibit orthologous Cas9 within the same subtype (15–18, 20), we tested *Neisseria* representatives of the three other type II-C Acr families (AcrIIC1*_Nme_*, AcrIIC2*_Nme_*, and AcrIIC3*_Nme_*) for inhibition of these two newly characterized Cas9 proteins. We found that all three of these previously characterized Acrs inhibit the DNA cleavage activity of both HpaCas9 and SmuCas9 (Fig. 3A).

10.1128/mBio.02321-18.3 FIG S3 Multiple sequence alignment of type II-C Cas9 proteins. Sequences of Cas9 proteins from N. meningitidis (C9X1G5), C. jejuni (WP_002924243.1), G. stearothermophilus (KZE96909.1), H. parainfluenzae (WP_049372626.1), and S. muelleri (WP_002641950.1) are aligned using MAFFT. Download FIG S3, PDF file, 1.4 MB. Copyright © 2018 Lee et al. 2018 Lee et al. This content is distributed under the terms of the Creative Commons Attribution 4.0 International license.

To further characterize the physical interactions of AcrIIC4*_Hpa_* and AcrIIC5*_Smu_* with HpaCas9 and SmuCas9, we coexpressed each 6×His-tagged Cas9 together with untagged Acr proteins in E. coli. Using Ni^2+^-affinity chromatography, we determined that AcrIIC4*_Hpa_* directly bound HpaCas9 and SmuCas9 (Fig. 3B). This is similar to the results observed for the previously characterized type II-C Acrs, which are known to bind to NmeCas9 (15, 20). In contrast, AcrIIC5*_Smu_* did not copurify with any of the tested Cas9 proteins under these conditions (Fig. 3B).

Previous work has shown that some Acrs, such as AcrIIC1 family members, inhibit Cas9s from Campylobacter jejuni (CjeCas9) and Geobacillus stearothermophilus (GeoCas9), in addition to NmeCas9 (20). CjeCas9 shares 32% sequence identity with NmeCas9, and GeoCas9 shares 39% (Fig. 2A and Fig. S3). To determine the range of activity of AcrIIC4*_Hpa_* and AcrIIC5*_Smu_*, we tested their inhibitory effects on type II-C Cas9s that have been validated for mammalian genome editing. Despite the abilities of both AcrIIC4*_Hpa_* and AcrIIC5*_Smu_* to inhibit DNA cleavage by NmeCas9 *in vitro*, neither prevented target DNA cleavage by CjeCas9 or GeoCas9 (Fig. S2C).

To confirm these *in vitro* results, we also performed E. coli-based phage targeting assays to assess the ability of AcrIIC4*_Hpa_* and AcrIIC5*_Smu_* to inhibit the activity of the various Cas9 orthologs. In this assay, Cas9 expressed from a plasmid in E. coli with an sgRNA that targets phage Mu led to a reduction in phage titer of ∼10^6^ PFU/ml (Fig. 3C), and we confirmed the coexpression of each Acr protein (Fig. S4). AcrIIC4*_Hpa_* expression completely inhibited the activity of HpaCas9 and decreased the activity of NmeCas9 by ∼100-fold (Fig. 3C). Similarly, AcrIIC5*_Smu_* expression completely inhibited the activity of both NmeCas9 and HpaCas9 (Fig. 3C), allowing phage Mu to plaque robustly. We were unable to test inhibition of SmuCas9 activity in E. coli because it failed to interfere with phage Mu plaquing even in the absence of Acr proteins, perhaps due to compromised function *in vivo* with the noncognate NmeCas9 sgRNAs. Consistent with the *in vitro* results, neither AcrIIC4*_Hpa_* nor AcrIIC5*_Smu_* inhibited phage targeting by GeoCas9 or CjeCas9 while AcrIIC1 inhibited all four Cas9 orthologs (Fig. 3C). These results, together with the *in vitro* DNA cleavage assays (Fig. 1), indicate that AcrIIC4*_Hpa_* and AcrIIC5*_Smu_* exhibit cross-species inhibitor activity (based on NmeCas9 inhibition) but have a narrower inhibitory spectrum than AcrIIC1 (20).

10.1128/mBio.02321-18.4 FIG S4 Expression levels of the indicated Acr proteins in bacteria coexpressing Geo, Nme, Hpa, or Cje Cas9. The SDS-PAGE gel was stained with Coomassie Blue. Download FIG S4, PDF file, 15.2 MB. Copyright © 2018 Lee et al. 2018 Lee et al. This content is distributed under the terms of the Creative Commons Attribution 4.0 International license.

### AcrIIC4*_Hpa_* and AcrIIC5*_Smu_* inhibit NmeCas9-mediated genome editing in mammalian cells.

**FIG 4:**
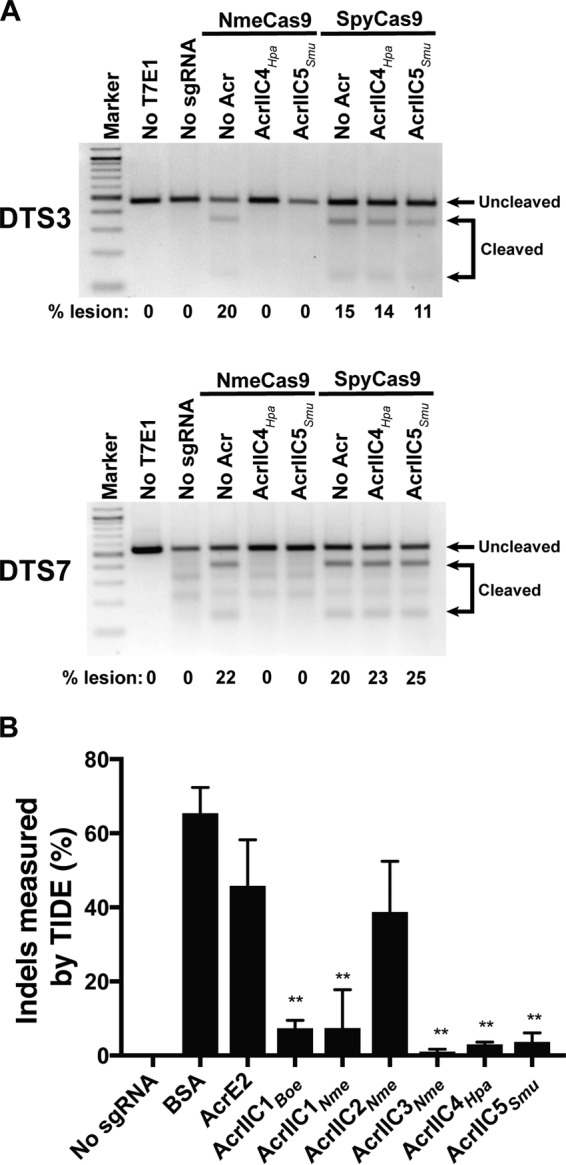
AcrIIC4*_Hpa_* and AcrIIC5*_Smu_* inhibit genome editing by NmeCas9 in human cells. (A) T7E1 assays of NmeCas9 or SpyCas9 editing efficiencies at two dual target sites (DTS3 and DTS7) upon transient plasmid transfection of human HEK293T cells. Constructs encoding anti-CRISPR proteins were cotransfected as indicated at the top of each lane. Mobilities of edited and unedited bands are indicated to the right, and editing efficiencies (“% lesion”) are given at the bottom of each lane. The figure is a representative of three replicates. (B) A bar graph of editing efficiencies measured by TIDE analysis upon RNP delivery of NmeCas9-sgRNA and Acr into HEK293T cells. Statistical significance was determined by two-tailed paired Student’s *t* test. Means and standard deviations from three biological replicates are indicated with lines (*, *P < *0.05; **, *P < *0.01; ***, *P < *0.001).

Validation of anti-CRISPR activity *in vitro* and in bacteria prompted us to test whether AcrIIC4*_Hpa_* and AcrIIC5*_Smu_* inhibit genome editing in mammalian cells. First, we used coimmunoprecipitation experiments to confirm that the NmeCas9/AcrIIC4*_Hpa_* physical interaction observed with recombinant proteins in E. coli (Fig. 3B) can also be detected in lysates from mammalian cells (Fig. S5A). Consistent with AcrIIC5*_Smu_* inhibition of NmeCas9 DNA cleavage activity *in vitro* (Fig. 1B), we also detected AcrIIC5*_Smu_*/NmeCas9 coimmunoprecipitation in mammalian lysates (Fig. S5A), even though purified, recombinant NmeCas9 did not pull down recombinant AcrIIC5*_Smu_* expressed in E. coli (Fig. 3B). To assess the inhibition of NmeCas9 genome editing, we cotransfected HEK293T cells transiently with plasmids expressing anti-CRISPR protein, NmeCas9 and sgRNAs targeting genomic sites. We then used T7 endonuclease 1 (T7E1) digestion to estimate genome editing efficiency. In agreement with our *in vitro* data, expression of AcrIIC4*_Hpa_* or AcrIIC5*_Smu_* reduced NmeCas9-mediated mutagenesis to undetectable levels at both tested sites (Fig. 4A). In contrast, they had no effect on genome editing at the same genomic sites by SpyCas9, which belongs to the type II-A CRISPR-Cas subtype and is very distantly related to NmeCas9 (Fig. 4A). Titration of plasmids expressing AcrIIC4*_Hpa_* or AcrIIC5*_Smu_* demonstrated potency against NmeCas9 that was comparable or superior to that of AcrIIC3*_Nme_* (Fig. S5B), which had previously been defined as the most potent NmeCas9 inhibitor in mammalian cells (15). For more rigorous quantitation of NmeCas9 editing, we used targeted deep sequencing at a distinct editing site (NTS1C) and detected little to no editing at higher doses of AcrIIC4*_Hpa_* or AcrIIC5*_Smu_* plasmids (Fig. S5C).

10.1128/mBio.02321-18.5 FIG S5 Anti-CRISPR proteins interact with NmeCas9 in mammalian cells to inhibit genome editing. (A) Anti-CRISPR proteins interact with NmeCas9 in HEK293T cells. Pulldowns of FLAG-tagged Acr and coimmunoprecipitated, HA-tagged NmeCas9 are confirmed by Western blotting. As a negative control, an untagged version of Acrs was used for pulldown. (B) T7E1 assays of NmeCas9 editing efficiencies at the DTS3 site upon transfection of HEK293T cells, with titrations of plasmids encoding AcrIIC4*_Hpa_* or AcrIIC5*_Smu_*. (C) Indel frequencies measured by targeted deep sequencing of PCR-amplified genomic DNA collected after transfection of NmeCas9 plasmid with or without Acrs at different dosages. Raw data are available in Data Set S1. (D) Abundance of steady-state anti-CRISPR proteins shown by Western blotting for FLAG-tagged Acr on the C terminus. GAPDH is used as a loading control. (E) Effect of anti-CRISPR proteins on the stability of NmeCas9 in the presence or absence of sgRNA in mammalian cells. Download FIG S5, PDF file, 3.1 MB. Copyright © 2018 Lee et al. 2018 Lee et al. This content is distributed under the terms of the Creative Commons Attribution 4.0 International license.

We previously noted a discrepancy in the potency of AcrIIC3*_Nme_*, which was least active in inhibiting N. meningitidis transformation but was most potent in cultured human cells (15). To address whether anti-CRISPR expression or stability correlates with inhibitory effect in mammalian cells, we estimated Acr protein abundance by Western blots using lysates from HEK293T cells transiently transfected with Acr expression plasmids (identical in all respects other than Acr ORF). Inhibition potency correlated well with the abundance of the anti-CRISPR, with AcrIIC4*_Hpa_* and AcrIIC5*_Smu_* showing the highest protein signal at steady state (Fig. S5D). To bypass the difference in protein abundance, we delivered a preformed ribonucleoprotein (RNP) complex of NmeCas9, sgRNA, and each Acr to HEK293T cells by electroporation. Then, we confirmed genome editing inhibition by AcrIIC4*_Hpa_* and AcrIIC5*_Smu_* using tracking of indels by decomposition (TIDE) analysis (34) (Fig. 4B). Acrs still displayed variations in activities even with RNP delivery, suggesting differences in protein stability, off-rate, or other intrinsic properties. Of note, however, AcrIIC4*_Hpa_* and AcrIIC5*_Smu_* consistently exhibited strong inhibitory potency both *in vitro* and in cultured cells (Fig. 1B and 4). Furthermore, AcrIIC4*_Hpa_* and AcrIIC5*_Smu_* coexpression increased the steady-state accumulation of NmeCas9 (with or without sgRNA coexpression), consistent with the possibility of a stabilizing physical interaction (Fig. S5E). Overall, these data demonstrate that the two new anti-CRISPRs directly bind to NmeCas9 and specifically inhibit its DNA cleavage activity in human cells.

### AcrIIC4*_Hpa_* and AcrIIC5*_Smu_* prevent stable DNA binding by NmeCas9.

**FIG 5:**
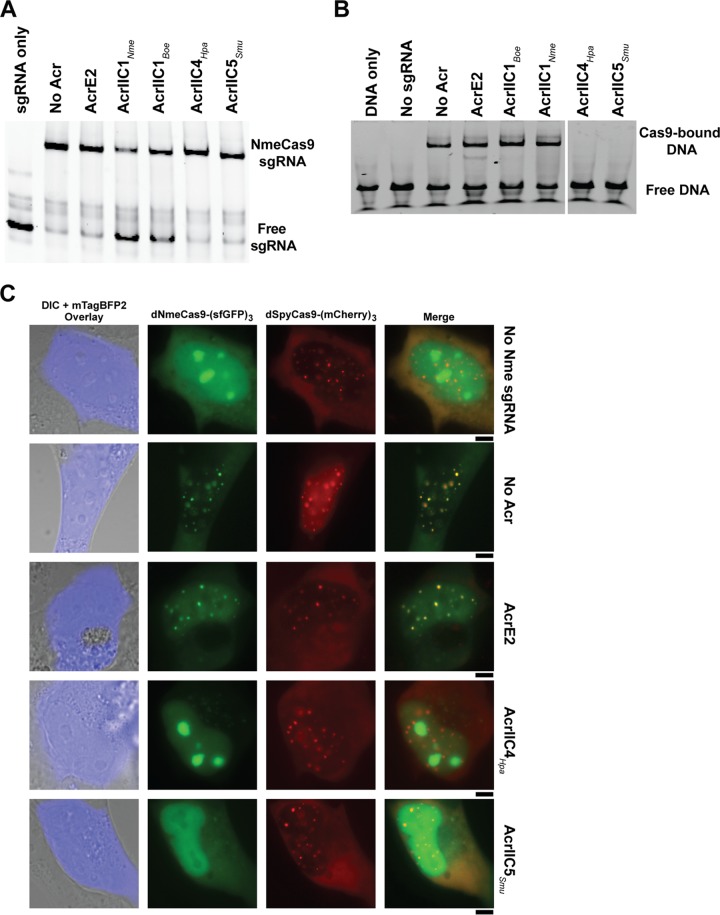
AcrIIC4*_Hpa_* and AcrIIC5*_Smu_* prevent stable DNA binding by NmeCas9. (A and B) A native gel of the sgRNA visualized by SYBR gold staining (A) and of the FAM-labeled target DNA (B), both of which were added last to NmeCas9 + Acr (and in panel B, + sgRNA) incubation. (C) Live-cell fluorescence images of U2OS cells transiently transfected with plasmids encoding dNmeCas9-(sfGFP)_3_, dSpyCas9-(mCherry)_3_, their respective telomeric sgRNAs, and Acrs. The plasmid encoding the Acr is also marked with the blue fluorescent protein, mTagBFP2, which is overlaid on a differential interference contrast (DIC) image of each cell. The specific version of each plasmid set (with or without sgRNAs, with or without anti-CRISPRs) is given to the right of each row. First column, differential interference contrast (DIC) and mTagBFP2 imaging, overlay. Second column, dNmeCas9-(sfGFP)_3_. Third column, dSpyCas9-(mCherry)_3_. Fourth column, dNmeCas9-(sfGFP)_3_ and dSpyCas9-(mCherry)_3_, merged. Scale bars, 5 µm.

Once we confirmed the anti-CRISPR inhibition of sgRNA-guided NmeCas9 DNA cleavage *in vitro* (Fig. 1) and genome editing in cells (Fig. 4), we then addressed the mechanisms of NmeCas9 inhibition by AcrIIC4*_Hpa_* and AcrIIC5*_Smu_*. Since structural and biochemical analysis of the anti-CRISPRs characterized to date suggests diverse and unique inhibitory mechanisms (11, 26, 27, 35), we tested multiple hypotheses: Acrs prevent sgRNA loading, DNA target binding (like AcrIIC3_*Nme*_ [15, 20]), or DNA target cleavage (like AcrIIC1_*Nme*_ [20]). First, we checked whether sgRNA loading onto NmeCas9 is inhibited by either anti-CRISPR. We carried out electrophoretic mobility shift assays (EMSAs) by incubating NmeCas9 and sgRNA with or without Acr, and then visualizing sgRNA mobility after native gel electrophoresis by SYBR Gold staining. In the absence of any anti-CRISPR, incubation of NmeCas9 with its cognate sgRNA resulted in a gel shift that indicates formation of a stable RNP complex (Fig. 5A). When NmeCas9 was incubated with a negative-control anti-CRISPR (AcrE2) before the addition of sgRNA, NmeCas9:sgRNA complex formation was unaffected. Similarly, when incubated with AcrIIC4*_Hpa_* and AcrIIC5*_Smu_*, efficient NmeCas9:sgRNA complex formation was again observed, suggesting that neither Acr protein significantly affected RNP assembly.

To test if target DNA engagement by the NmeCas9:sgRNA complex is prevented by either AcrIIC4*_Hpa_* or AcrIIC5*_Smu_*, we performed EMSAs and fluorescence polarization assays after incubating the RNP with each Acr, before adding target DNA (Fig. 5B and Fig. S5A). To inhibit DNA target cleavage, we omitted divalent metal ions from the reaction mixtures. While the target DNA exhibited the expected mobility shift in the absence of Acr, or in the presence of AcrE2 or AcrIIC1*_Nme_* (as expected [20]), both AcrIIC4*_Hpa_* and AcrIIC5*_Smu_* prevented NmeCas9 RNP binding to the target DNA. We also performed fluorescence polarization assays to measure the equilibrium binding constants of NmeCas9 RNP (0 to 2 µM) to target DNA (8 nM) in the presence or absence of Acrs (10 µM). As shown in Fig. S5A, AcrIIC4*_Hpa_* and AcrIIC5*_Smu_* significantly impair the DNA binding activity of NmeCas9:sgRNA, confirming our EMSA results. The measured *K_d_* of the NmeCas9 RNP to this target DNA (without Acr inhibition) is 86 ± 4 nM, similar to a previous measurement (70 ± 5 nM) with a different sgRNA/target site combination (20). The addition of AcrIIC4*_Hpa_* and AcrIIC5*_Smu_* reduced apparent DNA affinity by ∼9-fold (to 750 ± 150 nM) and ∼6-fold (to 450 ± 50 nM), respectively (Fig. S5A), similar to the ∼10-fold inhibition of NmeCas9 DNA binding by AcrIIC3*_Nme_* (20).

### AcrIIC4*_Hpa_* and AcrIIC5*_Smu_* are potent inhibitors of dNmeCas9-based tools in mammalian cells.

Many applications (e.g., CRISPRi and CRISPRa) have been developed for catalytically inactive (“dead”) Cas9 (dCas9) derivatives fused or tethered to various effector domains (36). To extend our findings from *in vitro* studies to mammalian cells, we tested whether AcrIIC4*_Hpa_* and AcrIIC5*_Smu_* prevent stable DNA binding of dNmeCas9 using previously established methods for live-cell imaging of telomeric foci. Briefly, transfection of plasmids expressing dCas9 orthologs fused to fluorescent proteins, as well as cognate sgRNAs targeting telomeres, enables telomeric foci to be visualized in U2OS cells (37). Orthogonal dNmeCas9-(sfGFP)_3_ and dSpyCas9-(mCherry)_3_ can be used in this fashion simultaneously to bind telomeres and generate colocalizing sfGFP and mCherry telomeric foci (38). Transfection of a third plasmid, marked with mTagBFP2 and encoding an anti-CRISPR protein, can be used to assess the anti-CRISPR’s effects on telomeric DNA binding in live cells (15). AcrE2 had no effect on telomeric foci formed by dNmeCas9-(sfGFP)_3_ and dSpyCas9-(mCherry)_3_, as seen previously (15); however, coexpression of AcrIIC4*_Hpa_* or AcrIIC5*_Smu_* resulted in loss of green foci formation by dNmeCas9-(sfGFP)_3_ without abolishing the red telomeric foci formed by dSpyCas9-(mCherry)_3_ (Fig. 5C). We then quantified the number of cells exhibiting telomeric dNmeCas9-(sfGFP)_3_ foci in a blinded experimental setup (Fig. S5B). We observed dNmeCas9-(sfGFP)_3_ foci in approximately 80% of cells in the absence of any Acr protein, in 70% of cells expressing AcrE2 protein (negative control), and in 0% of cells in the presence of AcrIIC3*_Nme_* (as a positive control [15, 20]) (Fig. S5B). We found that 0% of cells exhibited dNmeCas9-(sfGFP)_3_ telomeric foci when the two novel anti-CRISPRs were coexpressed (0 out of 78 for AcrIIC4*_Hpa_* and 0 out of 82 for AcrIIC5*_Smu_*) (Fig. S5B). These results confirm that AcrIIC4*_Hpa_* and AcrIIC5*_Smu_* inhibit stable DNA binding of dNmeCas9 in a cellular context, indicating their potential utility as potent off-switches for dNmeCas9-based applications.

These data from mammalian cells confirm the potential utility of AcrIIC4*_Hpa_* or AcrIIC5*_Smu_* (as well as other type II anti-CRISPR) proteins for modulating Cas9-dependent genome engineering applications across subtypes.

## DISCUSSION

**FIG 6:**
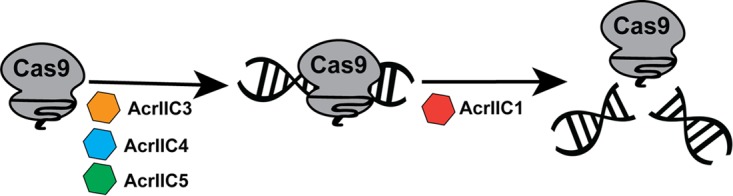
Summary of type II-C Cas9 orthologs and anti-CRISPR families. Type II-C anti-CRISPRs can act at distinct stages of Cas9-mediated target DNA cleavage. While AcrIIC1 binds to the HNH domain and inhibits a broad spectrum of Cas9 orthologs, AcrIIC4 and AcrIIC5 prevent DNA binding and have a narrower range of inhibition, similar to AcrIIC3.

The prevalence of CRISPR-Cas immune systems in bacteria and archaea has driven phages to evolve diverse anti-CRISPR proteins. Indeed, numerous anti-CRISPRs against type I and type II systems have been discovered in both bacteria and archaea since the first examples were reported in 2013 (10), with a range of inhibitory mechanisms for impairing Cas protein function (11, 26, 27, 35). Here, we report two new families of type II-C anti-CRISPR proteins, AcrIIC4*_Hpa_* and AcrIIC5*_Smu_*, and their cognate Cas9 proteins from H. parainfluenzae and S. muelleri. We define PAMs for the newly characterized HpaCas9 and SmuCas9 orthologs and show that they are functional *in vitro* and that HpaCas9 confers phage immunity in bacteria, expanding the functional Cas9 repertoire. These additional anti-CRISPRs and Cas9s total five anti-CRISPR families that differentially inactivate five different type II-C Cas9 orthologs (Fig. 6).

AcrIIC4*_Hpa_* and AcrIIC5*_Smu_* inhibit NmeCas9, HpaCas9, and SmuCas9 activity *in vitro*, as well as CRISPR interference activity in bacteria, and both also prevent NmeCas9-mediated genome editing in mammalian cells. The two new Acr families are the most potent among the type II-C Acrs, prevent substrate DNA binding by NmeCas9 and dNmeCas9, and exhibit higher specificities for inhibition of type II-C Cas9s in comparison to AcrIIC1 (20). AcrIIC4*_Hpa_* and AcrIIC5*_Smu_* activity was found to be specific to closely related Cas9 orthologs, as they did not inhibit the more distantly related CjeCas9 and GeoCas9 type II-C orthologs. Cross-species inhibitory effects of each Acr may be graded depending on the similarity of the Cas9 orthologs. Subtle differences may be sufficient to distinguish each anti-CRISPR’s breadth of inhibition as broad spectrum or highly specific, with gradations between these two extremes. For example, the AcrIIC1 family of Acrs can inhibit multiple Cas9s, likely because they bind to the highly conserved HNH domain (20), whereas other type II-C Acrs may bind to Cas9 domains that are less conserved (like the PID). The evolutionary pressure on Cas9s to evolve away from anti-CRISPR inhibition may promote diverse PAM specificities and other mechanistic distinctions between Cas9 orthologs. This may also explain why some hosts carry multiple, active CRISPR-Cas systems. Similarly, distinct anti-CRISPR specificities for inhibiting Cas9 orthologs could suggest different mechanisms of action. We show that AcrIIC4*_Hpa_* and AcrIIC5*_Smu_* prevent binding of Cas9 to target DNA, like AcrIIC3 and AcrIIA4 but unlike AcrIIC1 (15, 20). Target DNA binding could be prevented by precluding initial recognition of the PAM (similar to the strategy of AcrIIA4 [21–23]), by preventing one of the stages of R-loop formation and Cas9 structural rearrangement (39), or a combination of these.

Moreover, we have demonstrated the potential utility of Acr-mediated control of Cas9 and dCas9-based technologies by AcrIIC4*_Hpa_* and AcrIIC5*_Smu_*. Recently, AcrIIA4 (16) was used as an inhibitor of dSpyCas9 fused to a DNA demethylase, Tet1, to inactivate dSpyCas9-Tet1 DNA target binding (40). Separately, AcrIIA families were shown to prevent a gene-drive propagation in Saccharomyces cerevisiae (41). These are a few examples of the potential utility of Acrs as Cas9 off-switches. Many applications stand to benefit from increasing the numbers, specificities, and inhibitory mechanisms of anti-CRISPRs, for instance through combinatorial control over multiple Cas9/dCas9 proteins. For example, both broad-spectrum (e.g., AcrIIC1*_Nme_*) and highly specific (e.g., AcrIIC3*_Nme_*, -4*_Hpa_*, or -5*_Smu_*) anti-CRISPR proteins could be used to control multiple Cas9s simultaneously, or specific Cas9s but not others, upstream or downstream of target recognition, to achieve maximal flexibility of both genome manipulation and regulation.

Apart from potential uses in biotechnology, CRISPR-Cas systems and anti-CRISPR proteins that inactivate them are in strong accord with the Red Queen hypothesis, which proposes that bacteria must evolve new mechanisms to resist invaders while the invaders simultaneously evolve countermeasures (42). The widespread prevalence and extreme diversity of CRISPR-Cas systems in bacteria and archaea, as well as the adaptive nature of the resulting defenses, pose a significant challenge to phages and other MGEs. Anti-CRISPR proteins provide phages with an effective tactic to inactivate CRISPR-Cas systems and likely contribute to phage persistence in the face of host defense mechanisms. Many gaps remain in our understanding of the origins of these anti-CRISPRs and how they function in the context of phage predation. It is likely that these proteins have emerged independently and repeatedly through convergent evolution, as indicated by a lack of sequence or structural similarities among many reported Acrs (11, 26, 27, 35). A structural study of a capsid protein from a phage that infects Thermus thermophilus shares a common core β-barrel domain with AcrIIC1, suggesting an evolutionary source for an anti-CRISPR protein (43). Our ability to address these outstanding questions is limited by the relatively small number of examples of known anti-CRISPR proteins and their striking diversity in sequence and structures. Expanding the collection of diverse anti-CRISPR families and their cognate CRISPR effectors will help further our understanding of the arms race between phages and their hosts.

## MATERIALS AND METHODS

### Bioinformatics analysis for anti-CRISPR identification.

Putative anti-CRISPR genes were identified using the guilt-by-association bioinformatic method described previously (15). Briefly, BLASTp searches were conducted using *aca2* (WP_028357637.1) from B. oedipodis DSM 13743 (NZ_KK211205.1), and orthologs of *aca2* that had a small, uncharacterized hypothetical ORF immediately upstream were curated manually. The search yielded two high-confidence putative type II-C Acrs in strains of H. parainfluenzae 146_HPAR 254_56103_2121718_43__198__43_ (accession NZ_JVSL01000013.1) and S. muelleri ATCC 29453 (accession NZ_CP019448.1).

### Characterization of HpaCas9 and SmuCas9.

CRISPRfinder (http://crispr.i2bc.paris-saclay.fr) was used to identify the CRISPR locus of *H. parainfluenzae*. The spacers targeting the phage sequences were blasted via CRISPRTarget (http://bioanalysis.otago.ac.nz/CRISPRTarget) to predict the PAM present on the 3′ sequences. DNA and protein sequences of HpaCas9 and SmuCas9 orthologs are provided in Table S1 in the supplemental material.

### Plasmid construction.

Plasmids used in this study are described in Table S4.

10.1128/mBio.02321-18.10 TABLE S4 Strains and plasmids used in this study. Download Table S4, PDF file, 0.04 MB. Copyright © 2018 Lee et al. 2018 Lee et al. This content is distributed under the terms of the Creative Commons Attribution 4.0 International license.

### Cas9/sgRNA and anti-CRISPR vector for bacterial expression, protein purification, and *in vitro* transcription.

The pMCSG7-NmeCas9 expression vector and the sgRNA for *in vitro* transcription are as previously described (15). To make the HpaCas9 expression vector pEJS-MCSG7-HpaCas9, genomic DNA sequence from H. parainfluenzae DSM 8987 was obtained from DSMZ and cloned into the pMCSG7-NmeCas9 expression plasmid, replacing the NmeCas9 sequence using Gibson Assembly (NEB). The GeoCas9-expressing plasmid (expressing the GeoCas9 ortholog from G. stearothermophilus strain ATCC 7953) was obtained from Addgene (catalog no. 87700) and similarly cloned into the pMCSG7 vector. To make GeoCas9 from G. stearothermophilus strain L300, a gBlock (IDT) containing the PID was used to replace the PID of GeoCas9 from G. stearothermophilus strain ATCC 7953. For construction of sgRNA scaffolds for HpaCas9 and GeoCas9, the tracrRNA was predicted by crRNA repeat complementarity as well as homology to the NmeCas9 tracrRNA. These sgRNA scaffolds were ordered as gBlocks (IDT) along with overhangs to clone into pLKO.1 plasmid (15, 44) using Gibson Assembly (NEB). The CjeCas9 sgRNA plasmid was used as previously reported (20, 45). All sgRNA scaffolds were used as the templates to create *in vitro*-transcribed sgRNAs.

DNA sequences encoding candidate anti-CRISPR proteins were synthesized and cloned into a pUC57 mini (AmpR) vector with an N. meningitidis 8013 Cas9 promoter sequence for bacterial work, as done previously for other anti-CRISPRs (15). For anti-CRISPR protein purification, the Acr insert was amplified and inserted into the pMCSG7 backbone by Gibson Assembly (NEB), resulting in pMCSG7-Acr. Table S1 contains the DNA and protein sequences of the anti-CRISPRs tested in this study.

### Cas9/sgRNA and Acr vectors for mammalian expression.

For editing of genomic dual target sites by both SpyCas9 and NmeCas9, we used Cas9 and cognate sgRNA expression vectors that were described previously (15). To generate the Acr expression vector, the Acr ORF was amplified from pUC57-Acr and inserted into XhoI-digested pCSDest2 by Gibson Assembly (NEB).

### Vectors for fluorescence microscopy.

pHAGE-TO-DEST dSpyCas9-(mCherry)_3_ and dNmeCas9-(sfGFP)_3_ plasmids (38) were purchased from Addgene (catalog no. 64108 and 64109, respectively) and used directly for no-sgRNA control experiments. dNmeCas9-(sfGFP)_3_ and dSpyCas9-(mCherry)_3_ all-in-one plasmids have been described previously (15). To make Acr plasmids, we amplified an mTagBFP2 cassette and incorporated it into pCSDest2 vectors expressing the respective Acr by Gibson Assembly (NEB).

### Expression and purification of Acr and Cas9 proteins.

The expression and purification of Acrs and Cas9s were performed as described previously (7, 15). 6×His-tagged anti-CRISPRs and Cas9s were expressed in E. coli strain BL21 Rosetta(DE3). Cells were grown in LB or 2× YT medium at 37°C to an optical density (OD_600_) of 0.6 in a shaking incubator. At this stage the bacterial cultures were cooled to 18°C, and protein expression was induced by adding 1 mM IPTG. Bacterial cultures were grown overnight at 18°C (∼16 h), after which cells were harvested and resuspended in lysis buffer (50 mM Tris-HCl [pH 7.5], 500 mM NaCl, 5 mM imidazole, 1 mM DTT) supplemented with 1 mg/ml lysozyme and protease inhibitor cocktail (Sigma). Cells were lysed by sonication, and the supernatant was then clarified by centrifugation at 18,000 rpm for 30 min. The supernatant was incubated with preequilibrated Ni-NTA agarose (Qiagen) for 1 h. The resin was then washed twice with wash buffer (50 mM Tris-HCl [pH 7.5], 500 mM NaCl, 25 mM imidazole, 1 mM DTT). The proteins were eluted in elution buffer containing 300 mM imidazole. For Acr proteins, the 6×His tag was removed by incubation with His-tagged tobacco etch virus (TEV) protease overnight at 4°C followed by a second round of Ni-NTA purification to isolate successfully cleaved, untagged anti-CRISPRs (by collecting the unbound fraction). Cas9s were further purified using cation exchange chromatography using a Sepharose HiTrap column (GE Life Sciences). Size exclusion chromatography was used to purify NmeCas9 further in 20 mM HEPES-KOH (pH 7.5), 300 mM KCl and 1 mM TCEP.

### *In vitro* DNA cleavage.

For the *in vitro* DNA cleavage experiments with NmeCas9 (Fig. 1B and Fig. S1), NmeCas9 sgRNA targeting NTS4B was generated by *in vitro* T7 transcription (NEB). NmeCas9 (150 nM) was incubated with purified, recombinant anti-CRISPR protein (0 to 5 µM) in cleavage buffer (20 mM HEPES-KOH [pH 7.5], 150 mM KCl, 1 mM DTT) for 10 min. Next, sgRNA (1:1, 150 nM) was added and the mixture was incubated for another 15 min. Plasmid containing the target protospacer NTS4B was linearized by ScaI digestion. Linearized plasmid was added to the Cas9/sgRNA complex at 3 nM final concentration. The reaction mixtures were incubated at 37°C for 60 min, treated with 1 U proteinase K (NEB) at 50°C for 10 min, and visualized after electrophoresis in a 1% agarose/1× TAE gel.

### Phage immunity assay.

Plasmids expressing Cas9 targeting E. coli phage Mu were cotransformed into E. coli strain BB101 with plasmids expressing the anti-CRISPRs (20). Cells carrying both plasmids were grown in lysogeny broth (LB) supplemented with streptomycin (50 µg/ml) and chloramphenicol (34 µg/ml). Anti-CRISPR gene expression was induced using 0.01 mM IPTG for three hours. A lawn of 200 μl of cells in top agar was applied to LB agar plates supplemented with streptomycin, chloramphenicol, 200 ng/ml anhydrotetracycline (aTc), 0.2% arabinose ± 200 ng/ml aTc, and 10 mM MgSO_4_. Tenfold serial dilutions of phage Mu were spotted on top of the lawn, and the plates were incubated overnight at 37°C. To confirm the expression levels of the anti-CRISPR proteins in this assay, 500-µl aliquots of cells applied to the top agar were pelleted by centrifugation, resuspended in 100 µl of SDS-PAGE loading buffer, and run on a 15% Tris-Tricine gel, and the resulting protein gel was visualized by Coomassie blue (Bio-Rad).

### Cas9-Acr copurification.

Cas9 proteins were expressed from plasmid pMCSG7 with an N-terminal 6×His affinity tag in E. coli Rosetta cells. Untagged Acrs were coexpressed in the same cells from plasmid pCDF1b. Cells were grown in LB to an OD_600_ of 0.8, and protein production was induced with 2 mM IPTG overnight at 16°C. Cells were collected by centrifugation, resuspended in binding buffer (20 mM Tris, pH 7.5, 250 mM NaCl, 5 mM imidazole), and lysed by sonication, and cellular debris was removed by centrifugation. The cleared lysates were applied to Ni-NTA columns, washed with binding buffer supplemented with 30 mM imidazole, and eluted with 300 mM imidazole. Protein complexes were analyzed by SDS-PAGE followed by Coomassie staining.

### PAM determination.

A library of a protospacer with randomized PAM sequences was generated using overlapping PCRs, with the forward primer containing the 10-nt randomized sequence flanking the protospacer. The library was subjected to *in vitro* cleavage by purified recombinant HpaCas9 or SmuCas9 proteins as well as *in vitro*-transcribed sgRNAs. Briefly, 300 nM Cas9:sgRNA complex was used to cleave 300 nM target fragment in 1× reaction buffer (NEBuffer 3.1) at 37°C for 60 min. The reaction mixture was then treated with 1 U proteinase K (NEB) at 50°C for 10 min and run on a 4% agarose gel with 1× TAE. The segment of a gel where the cleavage products were expected to be was purified and subjected to library preparation as described previously (46). The library was sequenced using the Illumina NextSeq500 sequencing platform and analyzed with custom scripts.

### Electrophoretic mobility shift assay (EMSA).

NmeCas9 (1 µM) was incubated with 1 µM sgRNA in 1× binding buffer (20 mM Tris-HCl [pH 7.5], 150 mM KCl, 2 mM EDTA, 1 mM DTT, 5% glycerol, 50 µg/ml heparin, 0.01% Tween 20, 100 μg/ml BSA) for 20 min at room temperature to form the RNP complex. Acrs were added to a final concentration of 10 µM and incubated for an additional 20 min. Finally, the FAM-tagged NTS4B protospacer oligonucleotide was added to the mixture and incubated at 37°C for 1 h. The mixture was loaded onto a native 6% acrylamide gel, and the FAM-tagged DNA was visualized using a Typhoon imager.

### sgRNA EMSA.

NmeCas9 (1.5 µM) and anti-CRISPR (20 µM) proteins were preincubated in 1× binding buffer for 10 min, and then sgRNA (0.15 µM) was added to the reaction mixture for an additional 10 min. The complexes were resolved on a 6% polyacrylamide native gel, stained by SYBR Gold (ThermoFisher), and visualized with a Typhoon imager.

### Mammalian genome editing.

Plasmids for mammalian expression of NmeCas9, SpyCas9, their respective sgRNAs, and the anti-CRISPR proteins are listed in Table S4. Plasmid transfections, collection of genomic DNA, and T7E1 digestions were as described previously (15).

### Genome editing by Cas9 ribonucleoprotein (RNP) delivery.

RNP delivery of NmeCas9 was performed using a Neon electroporation system following the manufacturer’s instructions (ThermoFisher). Briefly, in a 10 μl reaction volume, 15 pmol of NmeCas9 and 150 pmol of anti-CRISPR protein were mixed in buffer R and incubated at room temperature for 20 min. Then, 20 pmol of T7 *in vitro*-transcribed sgRNA was added to the Cas9-Acr complex and incubated at room temperature for 30 min. Approximately 50,000 to 100,000 cells were mixed with the RNP-Acr-sgRNA complex, electroporated (Neon nucleofection system), and then plated in 24-well plates. Genomic DNA was extracted 48 h post-nucleofection using a DNeasy Blood and Tissue kit (Qiagen) according to the manufacturer’s protocol. Quantification of editing (% of amplicons exhibiting lesions) was done using TIDE analysis (34). PCR products spanning the target site were amplified using 2× Q5 master mix (NEB) and column-purified (Zymo). Purified amplicons were sent for Sanger sequencing (Genewiz), and trace files were analyzed by TIDE.

### Fluorescence microscopy of dNmeCas9.

Experimental procedures were as described previously (15). Briefly, U2OS cells were cotransfected with all-in-one plasmids (150 ng of each dNmeCas9 and dSpyCas9 plasmid), additional sgRNA-expressing plasmid, and 100 ng of anti-CRISPR/mTagBFP2 plasmid using PolyFect (Qiagen) according to the manufacturer’s instructions. After 24 h of incubation, live cells were imaged with a Leica DMi8 microscope equipped with a Hamamatsu camera (C11440-22CU), a 63× oil lens objective, and Microsystems software (LASX). Further imaging processing was done with Fiji-ImageJ. For quantification, only cells that exhibited mTagBFP2 and sfGFP fluorescence as well as dSpyCas9-(mCherry)_3_ telomeric foci were assessed for the presence or absence of colocalizing dNmeCas9-(sfGFP)_3_ telomeric foci.

### Fluorescence polarization.

For fluorescence polarization assays, preformed RNP complex of NmeCas9 and sgRNA was added to 1× binding buffer (20 mM Tris-HCl [pH 7.5], 150 mM KCl, 5 mM EDTA, 5 mM MgCl_2_, 1 mM DTT, 5% [vol/vol] glycerol, 50 μg/ml heparin, 0.01% Tween 20, and 100 μg/ml BSA) and incubated for 30 min followed by the addition of 10 μM Acrs. This mixture was incubated for 30 min followed by the addition of 8 nM FAM-tagged NTS4B protospacer (34 bp containing only 8-bp PAM duplex). After an incubation of 30 min the polarization measurements were made on Victor3 multilabel plate counters (Perkin Elmer). To calculate fraction-bound values, data were normalized by setting the lowest anisotropy to 0 and highest to 1. The curve fitting was performed in GraphPad Prism using the following equation:

$$Y=\frac{\left(\left|DNA\right|+\left|RNP\right|+K_{d}\right)-\sqrt{{\left(\left|DNA\right|+\left|RNP\right|+K_{d}\right)}^{2}-(4\times \left|DNA\right|\times \left|RNP\right|)}}{2\times |DNA|}$$

### Coimmunoprecipitation.

Plasmids expressing NmeCas9 and each anti-CRISPR protein were cotransfected into HEK293T cells. After 48 h, cell lysates were collected and bound to M2 FLAG magnetic beads (Sigma) overnight at 4°C. The beads were washed 5 times before elution by boiling with sample buffer (125 mM Tris-HCl, pH 6.8, with 4% SDS, 20% [vol/vol] glycerol, and 0.004% bromphenol blue). Subsequent Western blotting was performed as described below.

### Western blots.

For estimating anti-CRISPR protein levels in cells, plasmids encoding each Acr were transiently transfected into HEK293T cells using Polyfect (Qiagen). After 72 h, cell lysate was collected with lysis buffer (50 mM Tris-HCl [pH 7.4], 150 mM NaCl, 1 mM EDTA, 1% Triton X-100, 1× protease inhibitor cocktail [Sigma]). Lysates were boiled with the sample buffer supplemented with 5% 2-mercaptoethanol at 95°C for 5 min before running on 15% SDS-PAGE gels (Bio-Rad). Proteins were then transferred onto a PVDF membrane on a semidry transfer blot using the manufacturer’s instructions (Bio-Rad). Membranes were blocked in 5% dry milk, incubated with 1:5,000 primary antibodies (anti-FLAG raised in rabbit; Sigma) overnight, washed three times with TBST (25 mM Tris-HCl [pH 7.6], 125 mM NaCl, 1% Tween 20) for 5 min, and then incubated with HRP-conjugated secondary antibodies for detection with X-ray film (Kodak). For the NmeCas9 stability experiment, 150 ng of Cas9-expressing and 150 ng of sgRNA-expressing plasmids were transiently transfected with an additional 100 ng of Acr-expressing plasmid. For the no-sgRNA control, 150 ng of empty vector was used. Cell lysates were collected and run on a 6% SDS-PAGE gel as described above. After transfer and blocking steps, membranes were incubated with 1:5,000 anti-HA (mouse; Sigma) antibodies overnight and washed with TBST three times for 5 min before incubation with secondary antibodies (ThermoFisher) for 1 h. As a loading control, 1:5,000 anti-GAPDH (rabbit; Abcam) primary antibodies and HRP-conjugated secondary antibodies against rabbit (Bio-Rad) were used.

### Targeted deep sequencing analysis.

Targeted deep sequencing analyses were done as previously described (44). Briefly, we used a two-step PCR amplification approach to produce DNA fragments for each on-target and off-target site. In the first step, we used locus-specific primers bearing universal overhangs with ends complementary to the TruSeq adaptor sequences (Data Set S1). DNA was amplified with High Fidelity 2× PCR Master Mix (NEB) using appropriate annealing temperatures for the on-target (NTS1C) and off-target (NTS1C-OT1) sites. In the second step, the purified PCR pool was amplified with a universal forward primer and an indexed reverse primer to reconstitute the TruSeq adaptors. Full-size products (∼250 bp in length) were extracted using AMPure beads (Beckman Coulter). The purified library was deep sequenced using a paired-end 150 bp MiSeq run. Raw deep sequencing data and the results of statistical tests are reported in Data Set S1.

10.1128/mBio.02321-18.11 DATA SET S1 Raw deep sequencing data and the results of statistical tests. Download Data Set S1, XLSX file, 0.03 MB. Copyright © 2018 Lee et al. 2018 Lee et al. This content is distributed under the terms of the Creative Commons Attribution 4.0 International license.

### Data availability.

Raw data files are available upon reasonable request. High-throughput sequencing data are available at the NCBI Sequence Read Archive (accession no. PRJNA505886).

10.1128/mBio.02321-18.6 FIG S6 AcrIIC4*_Hpa_* or AcrIIC5*_Smu_* inhibit NmeCas9 before DNA binding. (A) Binding of NmeCas9 to partially duplexed DNA measured by fluorescence polarization assays with or without the indicated Acrs. The graph shows the average values (±SD) of three replicates. The curve was fitted to the equation shown in Materials and Methods, and the resulting *K_D_* values (nM) for AcrIIC5*_Smu_*, AcrIIC4*_Hpa_*, AcrIIC1*_Nme_*, and “No Acr” were 450.7 ± 47.6, 749.6 ± 157.7, 82.4 ± 6.5, and 85.9 ± 3.9, respectively. (B) Quantitation of dNmeCas9-(sfGFP)_3_ telomeric foci, as judged by colocalization with dSpyCas9-(mCherry)_3_ telomeric foci, in cells that express no anti-CRISPR, negative control anti-CRISPR (AcrE2), positive-control AcrIIC3*_Nme_*, AcrIIC4*_Hpa_*, or AcrIIC5*_Smu_*. Foci were scored blinded, i.e., without the experimenter knowing the sample identities. n, the number of cells that were scored under each condition over three biological replicates. Download FIG S6, PDF file, 1.0 MB. Copyright © 2018 Lee et al. 2018 Lee et al. This content is distributed under the terms of the Creative Commons Attribution 4.0 International license.
