# Supplementary figures and images for: Potent Cas9 Inhibition in Bacterial and Human Cells by AcrIIC4 and AcrIIC5 Anti-CRISPR Proteins
Source: mBio. 2018 Dec 4;9(6):e02321-18. doi: 10.1128/mBio.02321-18 (PMC6282205; doi:10.1128/mBio.02321-18)

Supplementary Figure 1

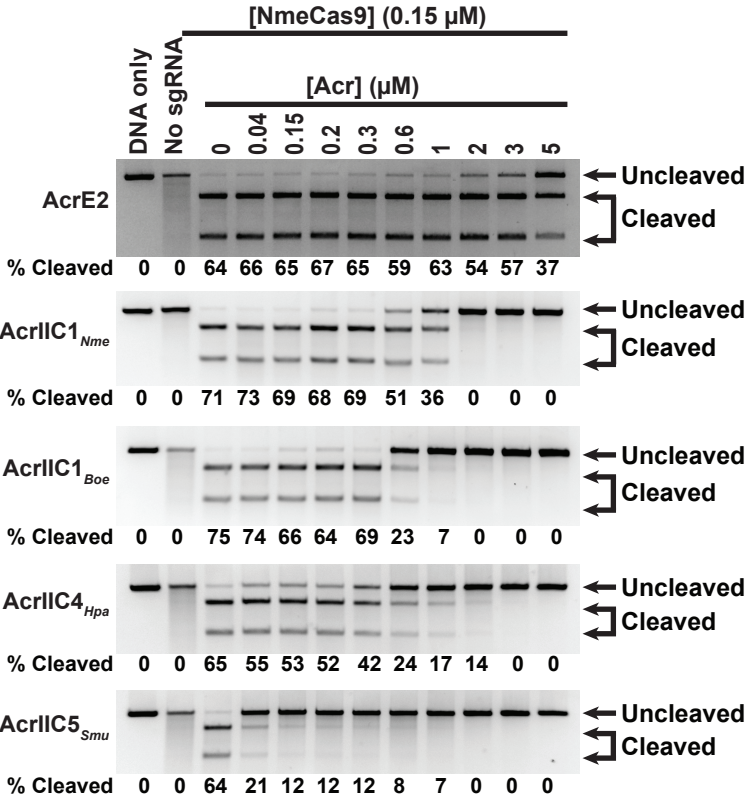

Supplement: FIG S1 [file mbo006184201sf1.pdf]

Supplementary Figure 3

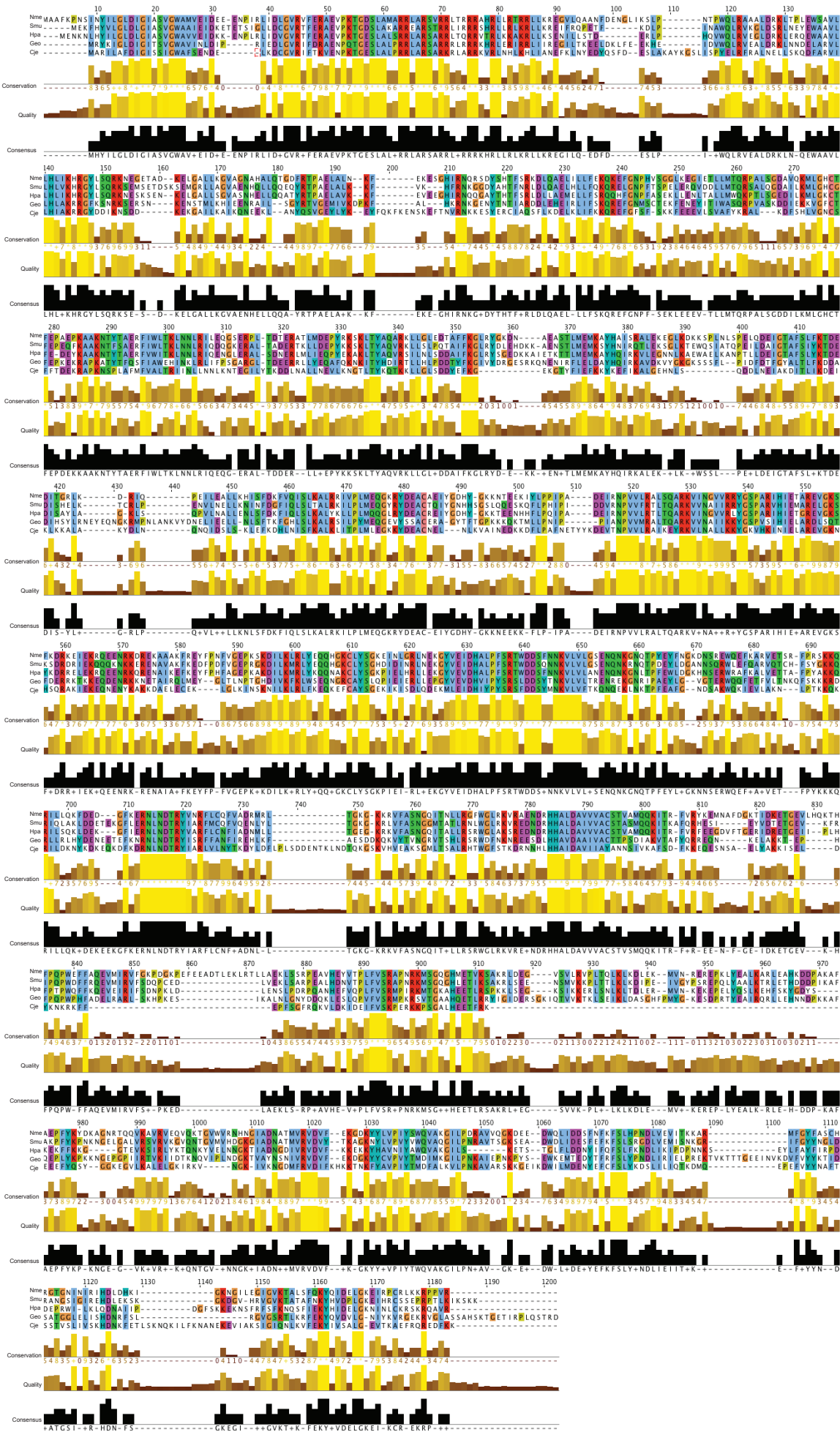

Supplement: FIG S3 [file mbo006184201sf3.pdf]

# Supplementary Figure 4

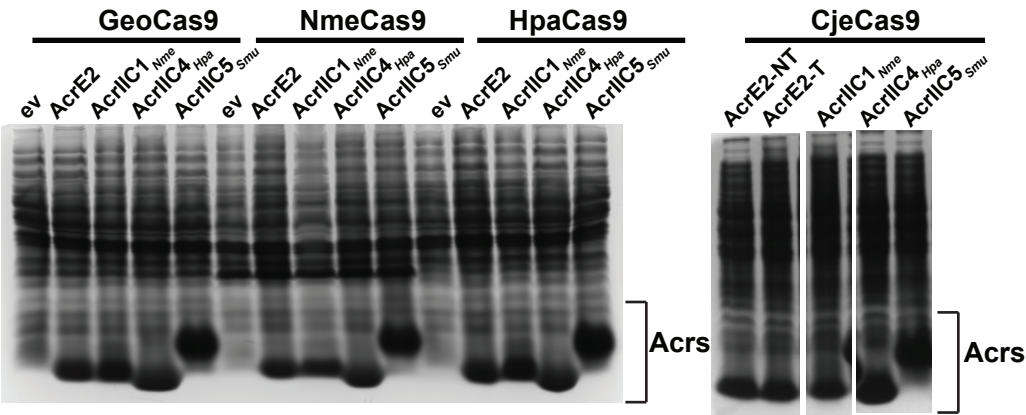

Supplement: FIG S4 [file mbo006184201sf4.pdf]

# Supplementary Figure 5

**A**

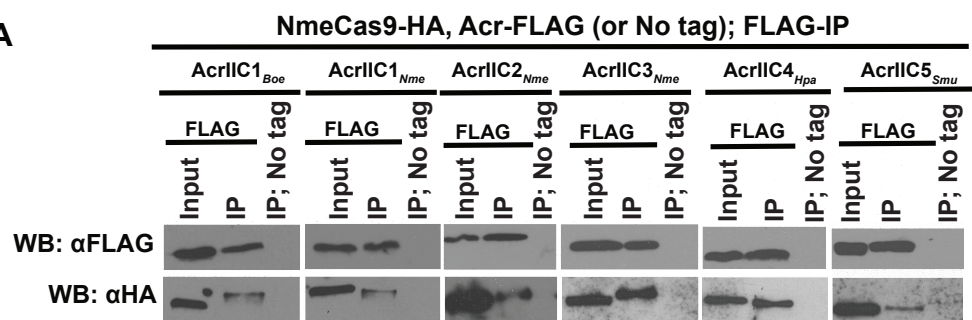

**B**

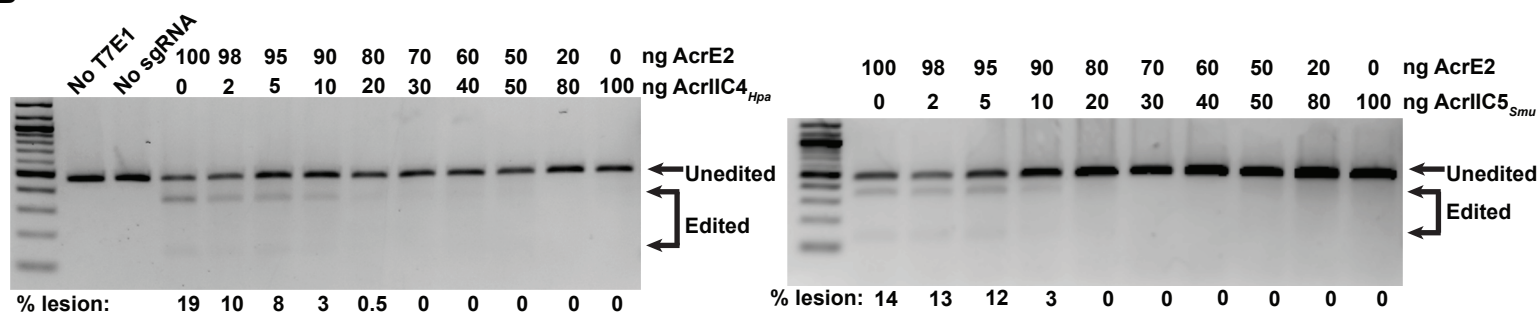

**C**

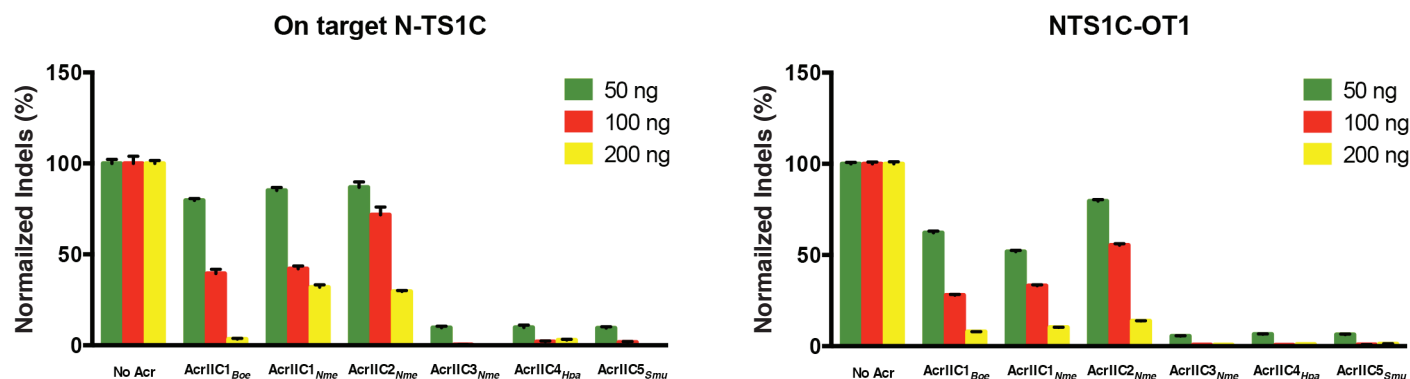

**D**

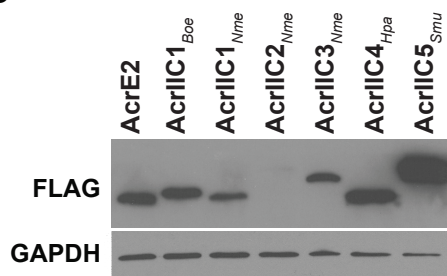

**E**

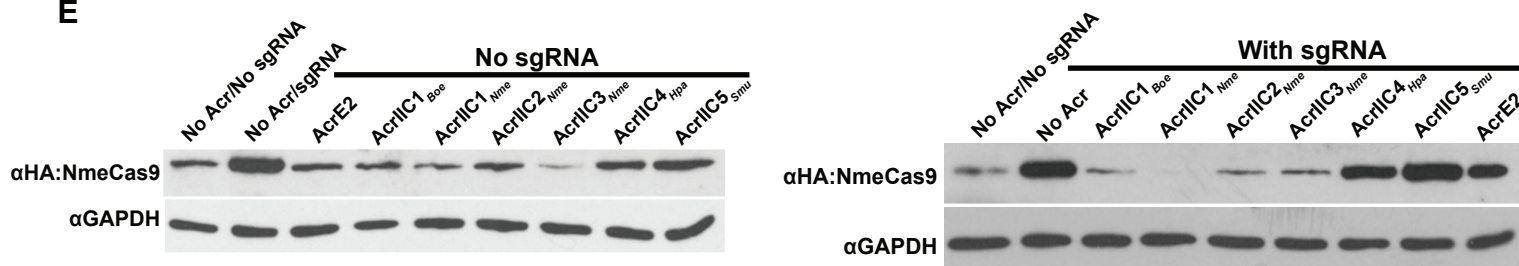

Supplement: FIG S5 [file mbo006184201sf5.pdf]

Supplementary Figure 6

A

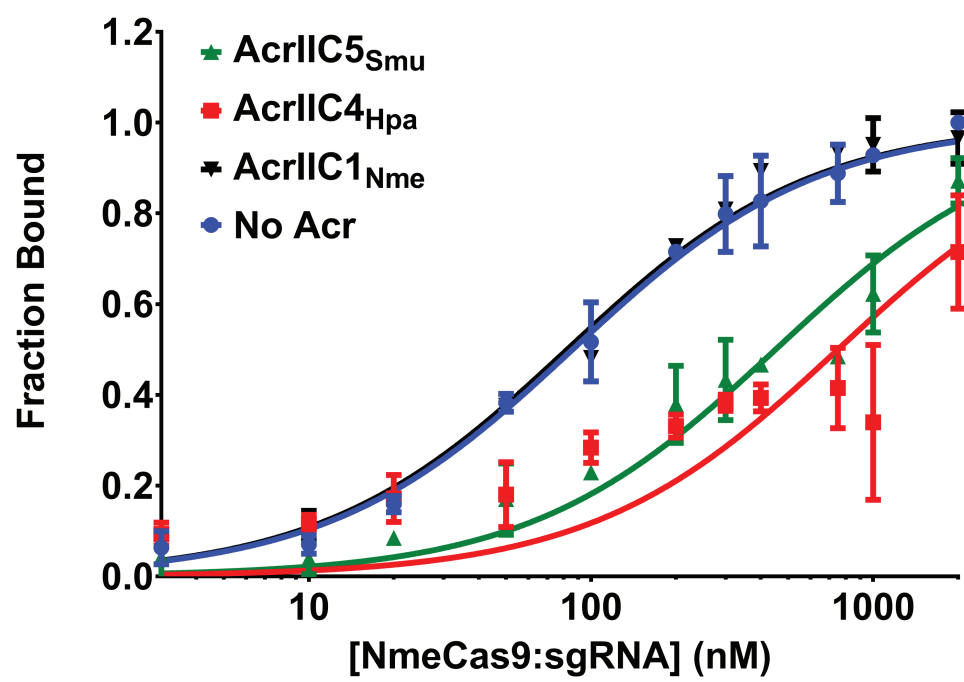

B

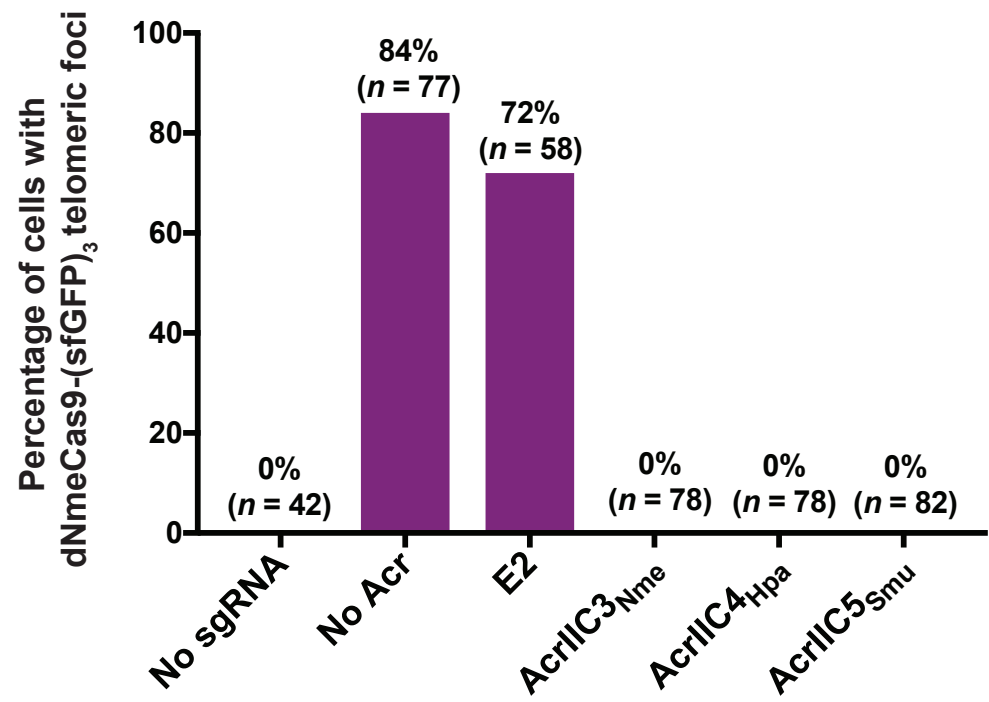

Supplement: FIG S6 [file mbo006184201sf6.pdf]
