# Supplementary material for: Potent Cas9 Inhibition in Bacterial and Human Cells by AcrIIC4 and AcrIIC5 Anti-CRISPR Proteins
Source: mBio. 2018 Dec 4;9(6):e02321-18. doi: 10.1128/mBio.02321-18 (PMC6282205; doi:10.1128/mBio.02321-18)
Supplement: TABLE S1 [file mbo006184201st1.pdf]

Supplementary Table 1: DNA and amino acid sequences of newly identified II-C anti-CRISPRs and Cas9 orthologs.

	Nucleotide sequence (5'→3')	Amino acid sequence (N→C)
AcrIIC4$_{Hpa}$	ATGAAGATCACCAGCAGCAACTTCGCGACCATTGCGACCAGCGAGAAC<br>TTTGCGAAGCTGAGCGTGCTGCCGAAAAACCACCGTGAGCCGATCAAG<br>GGTCTGTTCAAAAGCGCGGTTGAACAGTTTAGCAGCGCGCGTGACTTC<br>TTTAAGAACGAGAACTACAGCAAAGAGCTGGCGGAAAAGTTCAACAAAG<br>AAGCGGTGAACGAGGCGGTTGAAAAGCTGCAAAAAGCGATCGATCTGG<br>CGGAAAAACAGGGCATTCAATTT	MKITSSNFATIATSENFA<br>KLSVLPKNHREPIKGLFK<br>SAVEQFSSARDFFKNENY<br>SKELAEKFNKEAVNEAV<br>EKLQKAIDLAEKQGIQF
AcrIIC5$_{Smu}$	ATGAACAACAGCATCAAGTTCCACGTGAGCTACGACGGTACCGCGCGT<br>GCGCTGTTTAACACCAAGGAGCAGGCGGAAAAATACTGCCTGGTTGAG<br>GAAATTAACGATGAGATGAACGGCTATAAGCGTAAAAGCTGGGAGGAA<br>AAGCTGCGTGAGGAAAACTGCGCGAGCGTGCAGGACTGGGTTGAGAAG<br>AACTACACCAGCAGCTATAGCGACCTGTTCAACATCTGCGAGATTGAAG<br>TGAGCAGCGCGGGTCAACTGGTTAAGATCGACAACACCGAGGTGGACG<br>ATTTCGTTGAAAACTGCTATGGCTTTACCCTGGAGGACGATCTGGAGG<br>AATTCAACAAGGCGAAACAGTACCTGCAAAAATTTTATGCGGAGTGCGA<br>AAAC	MNNSIKFHVSYDGTARA<br>LFNTKEQAEKYCLVEEIN<br>DEMNGYKRKSWEEKLR<br>EENCASVQDWVEKNYTS<br>SYSDLFNICEIEVSSAGQL<br>VKIDNTEVDDFVENCYG<br>FTLEDDLEEFNKAKQYL<br>QKFYAECEN
HpaCas9	ATGGAAAATAAAAACTTAAATTATATCCTTGGTCTCGACCTTGGTATCG<br>CCTCTGTTGGCTGGGCTGTGGTTGAAATTGACGAAAAAGAGAATCCTC<br>TGCGTTTAATTGATGTTGGTGTACGTACTTTCGAACGAGCAGAAGTGC<br>CCAAAACAGGTGAAAGCCTTGCGCTTTCTCGCCGTTTAGCCCGCTCCG<br>CTCGTCGATTAACTCAACGTCGCGTTGCTCGTCTGAAAAAAGCAAAAC<br>GACTTCTAAAATCAGAAAATATCCTATTATCCACTGATGAACGTCTTCC<br>TCATCAAGTTTGGCAGCTACGTGTCGAAGGTCTAGATCATAAACTAGAA<br>CGCCAAGAATGGGCTGCTGTGTTATTACATTTAATTAAGCACCGTGGT<br>TATTTATCTCAGCGTAAAAATGAAAGTAAAAGCGAAAATAAAGAGCTAG<br>GTGCGTTATTAAGCGGTGTAGACAATAATCATAAATTACTGCAGCAAGC<br>TACATATCGCTCGCCAGCAGAACTTGCCGTAAAAAAATTTGAGGTCGAA<br>GAAGGTCATATCCGTAACCAACAAGGCGCCTATACTCACACGTTTAGCC<br>GACTAGACTTACTCGCCGAAATGGAACTCCTCTTCTCTCGTCAACAACA<br>CTTTGGTAATCCATTCGCTTCAGAAAAATTATTAGAAAATTTGACCGCA<br>CTTTTAATGTGGCAAAAACCTGCCTTATCTGGTGAAGCTATTTTAAAAA<br>TGCTCGGTAAATGTACTTTTGAAGATGAATATAAGGCAGCTAAGAACAC<br>TTATTCCGCAGAACGTTTTGTTTGGATAACGAAATTAAATAATTTACGT<br>ATTCAAGAAAATGGCTTAGAGCGTGCTTTAAATGATAATGAACGTTTAG<br>CATTAATGGAGCAACCTTATGACAAAAATAGATTATTCTATTCACAAGT<br>ACGCTCAATATTAAAATTATCTGATGAGGCAATCTTTAAAGGCCTCCGT<br>TATTCCGGTGAGGATAAAAAGGCCATTGAAACTAAAGCAGTACTGATG<br>GAAATGAAAGCCTATCACCAAATTCGTAAAGTATTGGAAGGTAATAACC<br>TAAAAGCTGAATGGGCAGAATTAAAGGCAAATCCAACATTATTAGATGA<br>AATTGGTACGGCATTTTCATTATATAAAACTGATGAAGATATCAGCGCT<br>TACTTAGCAGGAAAACTCTCTCAGCCTGTATTAAATGCCTTATTGGAAA<br>ATCTTAGTTTTGATAAATTTATCCAATTATCCCTTAAAGCTTTATATAA<br>ACTTCTACCATTAATGCAACAAGGACTACGCTATGATGAGGCTTGTCGT<br>GAAATTTATGGCGATCATTATGGTAAAAAAACAGAAGAAAACCATCACT<br>TCTTACCACAGATTCCAGCTGATGAAATCCGTAATCCTGTGGTTTTACG<br>AACACTCACGCAAGCTCGTAAAGTGATTAATGGCGTAGTGAGATTATAT<br>GGTTCGCCAGCTCGTATTCATATTGAAACGGGACGAGAAGTTGGCAAA<br>TCTTACAAAGATCGTCGTGAACTAGAAAAACGTCAGGAAGAAAATCGTA<br>AACAGCGTGAAAACGCAATCAAGGAATTTAAAGAGTACTTCCCTCATTT<br>TGCTGGTGAGCCTAAAGCCAAAGACATTTTAAAAATGCGACTTTATAAA<br>CAGCAAAATGCAAAATGTTTATATTCCGGCAAACCTATCGAATTGCACC<br>GTTTATTAGAAAAAGGCTATGTAGAAGTCGATCACGCCTTGCCGTTTT<br>CCCGTACTTGGGATGATAGTTTCAACAATAAAGTACTGGTGCTTGCTA<br>ATGAAAATCAAAACAAAGGCAATTTAACCCCTTTTGAATGGCTTGATGG<br>TAAACATAACAGCGAACGCTGGAGAGCGTTCAAAGCATTAGTTGAAACC<br>AGTGCATTCCCTTATGCGAAAAAACAACGCATCCTAAGCCAAAAACTTG<br>ATGAAAAGGGCTTTATTGAACGTAATTTAAATGATACGCGCTACGTTGC<br>TCGTTTCTTATGTAATTTTATTGCAGATAATATGCACTTAACAGGTGAA<br>GGAAAACGAAAAGTATTTGCTTCCAACGGGCAAATTACCGCTTTACTTC<br>GTAGCCGTTGGGGATTAGCAAAATCACGGGAAGACAATGACCGCCATC<br>ACGCCTTAGATGCCGTTGTGGTTGCTTGCTCAACCGTCGCCATGCAGC<br>AAAAAATCACACGTTTTGTTCGTTTTGAAGCTGGTGATGTATTCACTG<br>GTGAACGAATAGATCGTGAAACAGGTGAAATTATTCCATTACACTTCCC<br>TACTCCATGGCAATTTTTCAAACAAGAAGTTGAAATCCGAATTTTTAGT	MENKNLNYILGLDLGIAS<br>VGWAVVEIDEKENPLRLI<br>DVGVRTFERAEVPKTGE<br>SLALSRRLARSARRLTQR<br>RVARLKKAKRLLKSENIL<br>LSTDERLPHQVWQLRVE<br>GLDHKLERQEWAAVLL<br>HLIKHRGYLSQRKNESK<br>SENKELGALLSGVDNNH<br>KLLQQATYRSPAELAVK<br>KFEVEEGHIRNQQGAYT<br>HTFSRLDLLAEMELLFSR<br>QQHFGNPFASEKLLENLT<br>ALLMWQKPALSGEAILK<br>MLGKCTFEDEYKAAKNT<br>YSAERFVWITKLNNLRIQ<br>ENGLERALNDNERLALM<br>EQPYDKNRLFYSQVRSIL<br>KLSDEAIFKGLRYSGEDK<br>KAIETKAVLMEMKAYH<br>QIRKVLEGNNLKAEWAE<br>LKANPTLLDEIGTAFSLY<br>KTDEDISAYLAGKLSQPV<br>LNALLENLSFDKFIQLSLK<br>ALYKLLPLMQQGLRYDE<br>ACREIYGDHYGKKTEEN<br>HHFLPQIPADEIRNPVVL<br>RTLTQARKVINGVVRLY<br>GSPARIHIETGREVGKSY<br>KDRRELEKRQEENRKQ<br>RENAIKEFKEYFPHFAGE<br>PKAKDILKMRLYKQQNA<br>KCLYSGKPIELHRLLEKG<br>YVEVDHALPFSRTWDDS<br>FNNKVLVLANENQNKG<br>NLTPFEWLDGKHNSERW<br>RAFKALVETSAFPYAKKQ<br>RILSQKLDEKGFIERNLN<br>DTRYVARFLCNFIADNM<br>HLTGEGKRKVFASNGQI<br>TALLRSRWGLAKSREDN<br>DRHHALDAVVVACSTVA<br>MQQKITRFVRFEAGDVF<br>TGERIDRETGEIIPLHFPT<br>PWQFFKQEVEIRIFSDNP<br>KLELENRLPDRPQANHE<br>FVQPLFVSRMPTRKMTG<br>QGHMETVKSAKRLNEGI

	GATAATCCTAAACTGGAACTAGAAAATAGATTGCCGGATCGTCCACAAG<br>CCAATCATGAATTTGTCCAACCTCTGTTTGTATCTCGAATGCCAACACG<br>CAAAATGACCGGTCAAGGACACATGGAAACCGTGAAATCAGCGAAACG<br>TCTCAATGAAGGGATAAGCGTGATTAAAATGCCACTCACTAAATTAAAA<br>TTAAAAGATTTAGAATTGATGGTAAATCGTGAACGTGAAAAAGATCTTT<br>ATGATACTTTAAAAGCTCGTCTAGAGGCTTTTAATGATGATCCTGCTAA<br>AGCGTTTGCTGAACCTTTTATAAAAAAAGGTGGGGCTATTGTTAAATCA<br>GTACGAGTAGAACAAATACAAAAATCTGGCGTATTAGTCCGTGAGGGG<br>AATGGTGTCGCGGATAATGCCTCAATGGTGAGAGTGGATGTATTCACT<br>AAAGGTGGAAAATATTTCCTTGTGCCAATTTATACCTGGCAAGTCGCTA<br>AGGGAATTTTACCTAATAAAGCAGCAACACAATATAAAGATGAGGAAGA<br>TTGGGAAGTGATGGATAACTCTGCAACTTTCAAATTTTCATTACACCCA<br>AATGACTTAGTAAAATTAGTCACTAAAAAGAAAACCATTTTAGGTTACT<br>TTAATGGACTTAATCGTGCCACAGGTAATATAGATATTAAAGAACATGA<br>TTTAGATAAATCAAAAGGGAAACAAGGTATTTTTGAAGGTGTTGGTATT<br>AAATTAGCCCTTTCCTTCGAAAAATATCAAGTCGATGAACTTGGAAAAA<br>ATATTCGTTTATGTAAACCAAGTAAACGCCAACCTGTTCGTTAA	SVIKMPLTKLKLKDLEL<br>MVNREREKDLYDTLKA<br>RLEAFNDDPAKAFAEPFI<br>KKGGAIVKSVRVEQIQK<br>SGVLVREGNGVADNASM<br>VRVDVFTKGGKYFLVPI<br>YTWQVAKGILPNKAATQ<br>YKDEEDWEVMDNSATF<br>KFSLHPNDLVKLVTKKK<br>TILGYFNGLNRATGNIDI<br>KEHDLDKSKGKQGIFEG<br>VGIKLALSFEKYQVDELG<br>KNIRLCKPSKRQPVR
SmuCas9	ATGATGATGGAAAAATTTCACTATGTATTGGGTTTGGATTTGGGTATC<br>GCCTCTGTGGGGTGGGCTGCCATTGAAATTGACAAGGAAACCGAAACA<br>TCAATCGGTTTATTGGATTGCGGTGTCAGAACATTTGAACGTGCAGAA<br>GTACCCAAAACAGGCGATTCTCTTGCCAAAGCTCGCCGTGAAGCCAGA<br>AGTACTCGCCGTTTAATTCGCAGACGTTCGCATCGCTTATTACGTTTA<br>AAACGTTTATTGAAACGTGAAATTTTCAGGCAGCCTGAAACGTTTAAAG<br>ACTTACCAATCAATGCTTGGCAATTGCGTGTTAAAGGCTTGGATAGTC<br>GGTTGAATGAATATGAATGGGCGGCCGTTTTATTGCATTTGGTGAAGC<br>ATCGCGGTTATTTATCGCAACGCAAAAGCGAAATGAGCGAAACAGACA<br>GCAAATCTGAAATGGGCAGATTACTGGCAGGTGTGGCGGAAAATCACC<br>AACTTTTACAACAAGAACAATATCGTACACCAGCCGAATTAGCACTCAA<br>AAAATTTGTGAAACATTTTCGCAATAAAGGTGGCGATTATGCACACACT<br>TTCAACCGTTTGGATTTGCAAGCCGAATTGCATTTATTGTTTCAAAAAC<br>AACGTGAATTAGGCAATCCATTCACTTCACCAGAATTGGAACGGCAAGT<br>TGATGATTTGTTGATGACGCAGCGCAGTGCTTTACAAGGTGATGCGAT<br>TTTGAAAATGTTGGGTCATTGTGGGTTTGAACCTGAACAATTCAAAGC<br>AGCGAAAAACACATTCAGTGCCGAACGTTTTATTTGGTTGACAAAACTC<br>AATAATCTTCGCATTCAAGACCAAGGCAAAGAACGTGCGTTAACTGCCG<br>ATGAGCGTACCAAATTGTTGGACGAGCCTTATAAAAAAAGTAAATTGAC<br>TTACGCACAAGTTCGCAAATTATTAAGCTTGCCTCAAACTGCTATTTTT<br>AAAGGTTTGCGTTATGATTTGGAACATGACAAAAAAGCAGAAAACAGTA<br>CGTTGATGGAAATGAAATCCTATCACAACATCCGCCAAACATTGGAAAA<br>ATCAGGTTTGAAAACAGAATGGCAAAGTATTGCCACGCAGCCTGAAATT<br>TTAGATGCAATTGGCACGGCGTTTTCCATTTATAAAACCGATGAAGATA<br>TTTCGCATGAATTAAAAACGTGCAGGCTGCCTGAAAACGTATTGAATGA<br>ATTACTGAAAAACATCAATTTTGATGGATTCATTCAATTATCGTTGACT<br>GCATTACGCAAAATTTTGCCCTTGATGGAACAAGGCTACCGTTATGAT<br>GAAGCGTGTACCCAAATTTACGGTAATCATCATTCAGGCAGCTTGCAAC<br>AAGAATCAAAGCAATTTTTGCCACATATTCCGATTGATGATGTCCGAAA<br>TCCTGTGGTGTTCCGTACTTTGACCCAAGCAAGAAAAGTGGTGAATGC<br>GATTATTCGTCGGTATGGTTCGCCAGCTCGTGTGCATATTGAGATGGC<br>GCGTGAATTGGGTAAGTCTAAATCAGACCGTGACCGAATTGAAAAACAA<br>CAACAAAAAAATAAAAAAGAACGTGAAAACGCAGTCGCCAAATTCAAAG<br>AAGATTTCCCTGATTTTGTGGGCGAACCCAGAGGGAAAGATATTTTGA<br>AAATGCGTTTGTATGAACAACAACACGGCAAATGTTTGTATTCGGGTCA<br>TGATATTGATATTAATCGATTGAATGAAAAAGGTTATGTTGAAATTGAC<br>CATGCCCTGCCATTTTCACGGACTTGGGATGATAGTCAAAATAATAAAG<br>TTTTGGTACTTGGCAGCGAAAACCAAAATAAACGCAATCAAACGCCTGA<br>TGAATATTTGGACGGTGCAAACAATAGCCAACGTTGGCTTGAATTTCAA<br>GCGCGTGTACAAACTTGTCATTTTTCTTACGGTAAAAAACAACGCATTC<br>AATTAGCCAAATTAGACGATGAAACCGAAAAAGGATTTTTAGAACGCAA<br>TCTAAATGATACGCGGTATATTGCTCGTTTTATGTGTCAATTTGTCCAA<br>GAAAATTTATATTTGACAGGTAAAGGAAAACGTCTTGTTTTTGCATCAA<br>ACGGCGGAATGACCGCAACATTGAGAAATTTATGGGGTTTGAGAAAAG<br>TCCGTGAAGACAACGACCGCCATCATGCTCTTGATGCGATTGTGGTGG<br>CGTGTTCCACTGCTTCTATGCAACAAAAAATAACCAAAGCATTTCAACG<br>CCATGAAAGCATTGAATATGTGGATACCGAAACGGGCGAAGTAAAATTT<br>CGTATTCCACAGCCTTGGGATTTTTTCCGTCAGGAAGTGATGATTCGT<br>GTGTTCAGCGACCAACCGTGTGAAGATTTGGTAGAAAAATTGTCGGCT<br>CGTCCCGAAGCTTTGCATGACAACGTAACGCCTTTATTTGTCTCGCGT<br>GCACCAAATCGCAAAATGTCGGGGCAAGGGCATTTGGAAACCATCAAA<br>TCTGCAAAAAGGCTGTCTGAAGAAAACAGTATGGTTAAAAAACCATTAA<br>CCACATTGAAATTAAAAGATATTCCAGAAATCGTAGGCTACCCGAGTCG	MMMEKFHYVLGLDLGIA<br>SVGWAAIEIDKETETSIGL<br>LDCGVRTFERAEVPKTG<br>DSLAKARREARSTRRLIR<br>RRSHRLLRLKRLLKREIF<br>RQPETFKDLPINAWQLR<br>VKGLDSRLNEYEWAAVL<br>LHLVKHRGYLSQRKSEM<br>SETDSKSEMGRLLAGVA<br>ENHQLLQQEQYRTPAEL<br>ALKKFVKHFRNKGGDY<br>AHTFNRLDLQAELHLLF<br>QKQRELGNPFTSPELER<br>QVDDLLMTQRSALQGD<br>AILKMLGHCGFEPEQFK<br>AAKNTFSAERFIWLTKLN<br>NLRIQDQGKERALTADE<br>RTKLLDEPYKKSKLTYA<br>QVRKLLSLPQTAIFKGLR<br>YDLEHDKKAENSTLME<br>MKSYHNIRQTLEKSGLK<br>TEWQSIATQPEILDAIGT<br>AFSIYKTDEDISHELKTC<br>RLPENVLNELLKNINFDG<br>FIQLSLTALRKILPLMEQ<br>GYRYDEACTQIYGNHHS<br>GSLQQESKQFLPHIPIDD<br>VRNPVVFRTLTQARKVV<br>NAIIRRYGSPARVHIEMA<br>RELGKSKSDRDRIEKQQ<br>QKNKKERENAVAKFKE<br>DFPDFVGEPRGKDILKM<br>RLYEQQHGKCLYSGHDI<br>DINRLNEKGYVEIDHALP<br>FSRTWDDSQNNKVLVLG<br>SENQNKRNQTPDEYLDG<br>ANNSQRWLEFQARVQT<br>CHFSYGKKQRIQLAKLD<br>DETEKGFLERNLNDTRYI<br>ARFMCQFVQENLYLTGK<br>GKRLVFASNGGMTATLR<br>NLWGLRKVREDNDRHH<br>ALDAIVVACSTASMQQKI<br>TKAFQRHESIEYVDTET<br>GEVKFRIPQPWDFFRQE<br>VMIRVFSDQPCEDLVEK<br>LSARPEALHDNVTPLFVS<br>RAPNRKMSGQGHLETIK<br>SAKRLSEENSMVKKPLT<br>TLKLKDIPEIVGYPSREPQ<br>LYAALKTRLETHDDDPI<br>KAFAKPFYKPNKNGELG<br>ALVRSVRVKGVQNTGV

	TGAACCTCAATTGTATGCCGCATTGAAAACACGTTTAGAAACGCATGAT<br>GATGACCCAATTAAAGCCTTTGCCAAACCCTTTTACAAACCCAATAAAA<br>ATGGTGAATTGGGGGCGTTGGTTCGATCGGTGCGTGTGAAAGGTGTA<br>CAAAATACGGGTGTAATGGTTCATGATGGCAAAGGCATTGCCGATAAT<br>GCCACAATGGTTCGTGTTGATGTCTATACCAAAGCGGGCAAAAATTAC<br>CTTGTTCCTGTGTATGTTTGGCAGGTGGCTCAAGGAATTTTGCCAAAT<br>CGGGCGGTTACTTCTGGCAAAAGTGAAGCAGATTGGGATTTAATTGAT<br>GAAAGTTTTGAATTTAAATTTTCGCTGTCTCGTGGGGATTTAGTGGAA<br>ATGATTAGCAATAAAGGAAGAATTTTTGGTTATTACAATGGGTTAGATC<br>GTGCAAATGGAAGTATTGGGATTCGTGAACATGATTTGGAAAAGTCCA<br>AAGGAAAAGATGGTGTTCATCGTGTTGGCGTGAAAACCGCCACCGCAT<br>TCAACAAATACCACGTTGACCCACTTGGTAAAGAAATTCATCGGTGTTC<br>ATCTGAACCACGCCCCACATTAAAAATCAAATCCAAGAAATAA	MVHDGKGIADNATMVR<br>VDVYTKAGKNYLVPVYV<br>WQVAQGILPNRAVTSGK<br>SEADWDLIDESFEFKFSLS<br>RGDLVEMISNKGRIFGYY<br>NGLDRANGSIGIREHDLE<br>KSKGKDGVHRVGVKTA<br>TAFNKYHVDPLGKEIHR<br>CSSEPRPTLKIKSKK
