# Supplementary material for: Potent Cas9 Inhibition in Bacterial and Human Cells by AcrIIC4 and AcrIIC5 Anti-CRISPR Proteins
Source: mBio. 2018 Dec 4;9(6):e02321-18. doi: 10.1128/mBio.02321-18 (PMC6282205; doi:10.1128/mBio.02321-18)
Supplement: TABLE S2 [file mbo006184201st2.pdf]

Supplementary Table 2: AcrIIC5$_{Smu}$ homolog % identities.

	AcrIIC5$_{Smu}$	AcrIIC5$_{Nzo}$	AcrIIC5$_{N10023}$	AcrIIC5$_{N95_16}$	AcrIIC5$_{Nwa}$
S. muelleri	100				
N. zoodegmatis	28	100			
Neisseria sp. 10023	30	75	100		
Neisseria sp. N95_16	27	72	84	100	
N. wadsworthii	25	47	51	50	100
