# Supplementary material for: Potent Cas9 Inhibition in Bacterial and Human Cells by AcrIIC4 and AcrIIC5 Anti-CRISPR Proteins
Source: mBio. 2018 Dec 4;9(6):e02321-18. doi: 10.1128/mBio.02321-18 (PMC6282205; doi:10.1128/mBio.02321-18)
Supplement: TABLE S3 [file mbo006184201st3.pdf]

Supplementary Table 3: Pairwise percent protein identities between type II-C Cas9 orthologs.

	# of amino acids	NmeCas9	SmuCas9	HpaCas9	GeoCas9	CjeCas9
NmeCas9	1,082	100				
SmuCas9	1,063	62	100			
HpaCas9	1,049	59	55	100		
GeoCas9	1,087	39	39	40	100	
CjeCas9	984	32	31	32	32	100
