# Supplementary material for: Potent Cas9 Inhibition in Bacterial and Human Cells by AcrIIC4 and AcrIIC5 Anti-CRISPR Proteins
Source: mBio. 2018 Dec 4;9(6):e02321-18. doi: 10.1128/mBio.02321-18 (PMC6282205; doi:10.1128/mBio.02321-18)
Supplement: TABLE S4 [file mbo006184201st4.pdf]

Supplementary Table 4: Strains and plasmids used in this study.

**Bacterial strains**		**Source**
Escherichia coli Rosetta (DE3)		Thermo Fisher Scientific
Escherichia coli Bl21		A. Davidson Lab
Escherichia coli BB101		A. Davidson Lab
**Plasmids for protein purification and binding assays**		
pEJS658	pMCSG7-AcrE2	This study
pEJS659	pMCSG7-AcrIIC1Boe	This study
pEJS660	pMCSG7-AcrIIC1Nme	This study
pEJS661	pMCSG7-AcrIIC2Nme	This study
pEJS662	pMCSG7-AcrIIC3Nme	This study
pEJS663	pMCSG7-AcrIIC4Hpa	This study
pEJS664	pMCSG7-AcrIIC5Smu	This study
pEJS1026	pMCSG7-HpaCas9	This study
pEJS1027	pMCSG7-SmuCas9	This study
pEJS973	pMCSG7-NmeCas9	This study
pCDF-ACRE2		A. Davidson Lab
pCDF-ACRIIC1Nme		A. Davidson Lab
pCDF-ACRIIC4Hpa		This study
pCDF-ACRIIC5Smu		This study
**Plasmids expressing Cas9/sgRNA for phage Mu targeting assay**		
pBG_GeoCas9sgRNAMu		Harrington et al., *Cell,* 2017
pBG_HpaCas9sgRNAMu		This study
pBG_NmeCas9sgRNAMu		A. Davidson Lab
pBG_CjeCas9sgRNAMu		A. Davidson Lab
**Plasmids for mammalian genome editing**		
pEJS540	pCSDest2-AcrIIC4-Hpa	This study
pEJS542	pCSDest2-AcrIIC5-Smu	This study
pEJS581	pCSDest2-AcrIIC4Hpa-FLAG-NLS	This study
pEJS583	pCSDest2-AcrIIC5Smu-FLAG-NLS	This study
pEJS24	pCSDest2-SpyCas9-NLS-3XHA-NLS	Addgene # 69220
pEJS424	pCSDest2-NmeCas9-NLS-3XHA-NLS	Addgene #87448
pEJS427	pCSDest2-AcrE2	Addgene # 85677
pEJS430	pCSDest2-AcrIIC1Boe	Addgene #85678
pEJS433	pCSDest2-AcrIIC1Nme	Addgene #85679
pEJS436	pCSDest2-AcrIIC2Nme	Addgene #85712
pEJS443	pCSDest2-AcrIIC3Nme	Addgene #85713
pEJS333	pLKO.1-puro U6 Nme-sgRNA BfuAI stuffer	Addgene # 86195
pEJS334	pLKO.1-puro U6 Spy-sgRNA BfuAI stuffer	Addgene # 52628
pEJS15	pSimpleII-NmeCas9-sgRNA/Empty	E. Sontheimer Lab
**Plasmids for fluorescence imaging**		

pEJS333	pLKO.1-puro U6 Nme-sgRNA BfuAI stuffer	Addgene # 86195
pEJS334	pLKO.1-puro U6 Spy-sgRNA BfuAI stuffer	Addgene # 52628
pEJS466	pHAGE-TO-Nme dCas9-3xGFP	Addgene #64109
pEJS467	pHAGE-TO-Spy dCas9-3xmCherry	Addgene #64108
pEJS468	pLKO.1-NmeSgRNA/DTS13-Telomere	Addgene #85714
pEJS469	pLKO.1-SpySgRNA/DTS13-Telomere	Addgene #85715
pEJS476	pHAGE-TO-Nme dCas9 3XGFP-SgRNA/Telomere-All-in-one	Addgene #85716
pEJS477	pHAGE-TO-Spy dCas9 3XmCherry-SgRNA/Telomere-All-in-one	Addgene #85717
pEJS507	pCSDest2-noAcr-mTagBFP2-IRES	Addgene #85748
pEJS481	pCSDest2-AcrE2-mTagBFP2-IRES	Addgene #85749
pEJS482	pCSDest2-AcrIIC3Nme-mTagBFP2-IRES	Addgene #85750
pEJS592	pCSDest2-AcrIIC4Hpa-BFPv2-IRES	This study
pEJS593	pCSDest2-AcrIIC5Smu-BFPv2-IRES	This study
